# Paracrine secretion of IL8 by breast cancer stem cells promotes therapeutic resistance and metastasis of the bulk tumor cells

**DOI:** 10.1186/s12964-023-01068-6

**Published:** 2023-03-13

**Authors:** Mingming Wu, Xiao Zhang, Weijie Zhang, Linlin Yan, Xiangtian Liu, Min Zhang, Yueyin Pan, Peter E. Lobie, Xinghua Han, Tao Zhu

**Affiliations:** 1 Department of Oncology, The First Affiliated Hospital of USTC, Division of Life Sciences and Medicine, University of Science and Technology of China, Hefei, 230027 Anhui China; 2 The CAS Key Laboratory of Innate Immunity and Chronic Disease, Division of Life Sciences and Medicine, University of Science and Technology of China, Hefei, Anhui China; 3 Institute of Biopharmaceutical and Health Engineering, Tsinghua Berkeley Shenzhen Institute, Tsinghua Shenzhen International Graduate School, Shenzhen, 518055 China; 4 Institute of Biomedical Health Technology and Engineering, Shenzhen Bay Laboratory, Shenzhen, 518132 China

**Keywords:** Cancer stem cell, IL8, Bulk cancer cell, Tamoxifen resistance, Metastasis

## Abstract

**Background:**

Breast tumors consist of heterogeneous cellular subpopulations that differ in molecular properties and functional attributes. Cancer stem cells (CSCs) play pivotal roles in cancer therapeutic failure and metastasis. However, it remains indeterminate how CSCs determine the progression of the bulk cancer cell population.

**Methods:**

Co-culture systems in vitro and co-implantation systems in vivo were designed to characterize the interactions between breast cancer stem cells (BCSCs) and bulk cancer cells. RNA sequencing was performed to study the functional and mechanistic implications of the BCSC secretome on bulk cancer cells. A cytokine antibody array was employed to screen the differentially secreted cytokines in the BCSC secretome. Tail vein injection metastatic models and orthotopic xenograft models were applied to study the therapeutic potential of targeting IL8.

**Results:**

We identified that the BCSC secretome potentiated estrogen receptor (ER) activity in the bulk cancer cell population. The BCSC secretome rendered the bulk cancer cell population resistant to anti-estrogen and CDK4/6 inhibitor therapy; as well as increased the metastatic burden attributable to bulk cancer cells. Screening of the BCSC secretome identified IL8 as a pivotal factor that potentiated ERα activity, endowed tamoxifen resistance and enhanced metastatic burden by regulation of bulk cancer cell behavior. Pharmacological inhibition of IL8 increased the efficacy of fulvestrant and/or palbociclib by reversing tamoxifen resistance and abrogated metastatic burden.

**Conclusion:**

Taken together, this study delineates the mechanism by which BCSCs determine the therapeutic response and metastasis of bulk cancer cells; and thereby suggests potential therapeutic strategies to ameliorate breast cancer outcomes.

Video Abstract

**Supplementary Information:**

The online version contains supplementary material available at 10.1186/s12964-023-01068-6.

## Background

Cancer is hierarchically organized with a subpopulation of cancer stem cells (CSCs) at the apex [1]. As a rare cancer cell population characterized by the ability of tumor initiation and self-renewal [2, 3], CSCs share critical properties with normal embryonic or tissue stem cells, including stem cell-associated marker expression, differentiation and lineage generation [4]. The relative percentage of the CSC population has been observed to be clinically correlated with poor prognosis [5]. Thus, it is thought that CSCs, as a minority cell population, may determine the biological and pathological characteristics of cancer progression [6].

Cancer that relapses after an initial response to therapy develops persistent drug resistance [7]. To a large extent, cancer therapeutic failure has been attributed to the defect of conventional therapies which selectively eliminated the cancer bulk without ablation of CSCs [8]. CSCs are intrinsically resistant to chemotherapy and some targeted therapeutic approaches [9]. The CSC hypothesis espouses that CSCs that survive therapeutic approaches can support the re-establishment of the cancer [10]. Characterized by quiescence, an enhanced capacity for DNA repair, and high expression levels of ABC-transporters [8], the proportion of the CSC population in relapsing drug-resistant cancer is increased. Nevertheless, the proportion of CSCs is highly variable and the non-CSCs remain the majority of the drug-resistant cancer cell population [11–13]. Thus, the hypothesis that the inherent properties of CSCs alone provide the explanation for drug resistance is too simplistic. How the interaction between CSCs and the bulk cancer cells determines drug resistance in the bulk remains unappreciated.

Metastasis remains the predominant cause of death in breast cancer patients [14]. Metastatic potential was attributed to multiple factors that determine cancer cell invasion, survival, colonization and outgrowth [15], wherein CSCs play a critical role [16]. CSCs exhibit increased motility, a mesenchymal phenotype and resistance to various extrinsic stresses [17, 18]. However, whereas much attention has been paid to the intrinsic properties of CSCs in cancer metastasis, there is a paucity of knowledge of the interaction between CSCs and the bulk cancer cell population in invasion and the metastatic process. In this study, co-culture in vitro and co-implantation systems in vivo were employed to determine the role of interactions between BCSCs and bulk cancer cells in cancer response to treatment and in metastasis.

## Materials and methods

### Cell lines

The MCF-7, T47D and MDA-MB-231 cells were purchased from ATCC. SUM159 cells were obtained from Professor Suling Liu (Fudan University, China). HEK293T cells were obtained from Dr. Huanfeng Zhang (USTC). Cells were cryopreserved soon upon receipt and continuously cultured for less than 2 months. MCF-7, T47D and MDA-MB-231 cells have been authenticated by STR genotyping. No cross-contamination with other human cells was observed. Possible mycoplasma contamination of all cell lines is routinely and regularly monitored. MCF-7 and T47D cells were cultured in RPMI 1640 (Gibco, California, America) supplemented with 10% fetal bovine serum (Gibco, California, America) at 37 °C and 5% CO_2_. MDA-MB-231 cells were cultured in DMEM (Gibco) supplemented with 10% fetal bovine serum (Gibco, California, America) at 37 °C and 5% CO_2_. SUM159 cells were cultured in Ham’s F12 (Gibco, California, America) supplemented with 5% fetal bovine serum (Gibco, California, America), 5 μg/ml insulin (Sigma, Darmstadt, Germany) and 1 μg/ml hydrocortisone (Sigma, Darmstadt, Germany).

### Mice

The 4-week old female BALB/c nude mice were obtained from SLAC laboratory animals (Shanghai, China). The mice were housed in an SPF facility with a 12 h light–dark cycle at 22–24 °C. In MCF-7 and MCF-7 TamR xenograft models, a slow-release pellet containing 0.36 mg of 17β-estradiol (Innovative Research of America, Sarasota, USA) was subcutaneously implanted into the back of nude mice 3 days prior to the xenograft implantation. For orthotopic xenograft models, the cells suspended in PBS were mixed at 1:1 with Matrigel (Corning, USA). The mixed cells were injected into the second mammary fat pad of nude mice. Xenograft growth rates were analyzed by measuring tumor length (L) and width (W), and calculating volume through the use of the formula LW^2^/2. For tail vein injection, the cells suspended in PBS were injected into the lateral tail vein of nude mice. For tamoxifen treatment, a slow-release tamoxifen pellet (5 mg, 60-day release) was subcutaneously implanted into the back of nude mice, separated from the 17β-estradiol pellet. Repertaxin was administered twice-daily *s.c.* at a concentration of 15 mg/kg, control animals received vehicle. Fulvestrant (Vetter Pharma, Germany) was administered (5 mg/animal per week) via subcutaneous injection. Palbociclib (Targetmol, Massachusetts, USA) was administered (100 mg/kg/week) via orogastric gavage. In vivo bioluminescent imaging was performed to determine the xenograft growth and metastasis of luciferase labeled cells. Mice were injected *i.p.* with 150 μg/g of D-luciferin (12 mg/ml in PBS) and imaged 10 min after injection using a PerkinElmer IVIS Spectrum system.

### Reagents

IL8, ICAM1, MIF, CXCL12, EGF and bFGF recombinant protein were from PeproTech (Rocky Hill, America). Tamoxifen and Insulin were purchased from Sigma-Aldrich (Darmstadt, Germany). The Wnt/β-catenin pathway inhibitor XAV939 and activator CHIR99021 were from TargetMol (Massachusetts, America). The CXCR1 and CXCR2 antagonist Repertaxin was obtained from MedChem Express (America). The Annexin V-FITC Apoptosis Detection Kit (Cat#APOAF-50TST) was obtained from Sigma-Aldrich (Darmstadt, Germany). The ALDEFLUOR™ Kit (Cat#01700) was obtained from Stem Cell (Canada). ChIP Assay Kit (Cat#P2078) was obtained from Beyotime (Shanghai, China). Proteome Profiler Human Cytokine Array Kit (Cat#ARY005B) was purchased from R&D System (California, USA). Human IL-8/NAP-1 Platinum ELISA Kit (Cat#BMS204/3) was purchased from eBioscience (California, USA).

### Plasmid construction and transfection

The shRNA plasmids of *IL8* and *CTNNB1* were obtained from The RNAi Consortium (MISSION® TRC shRNA library, Sigma-Aldrich). For luciferase reporter plasmids containing the *IL8* promoter, the DNA fragment upstream of the *IL8* gene carrying the TCF4 binding site was cloned into the pGL3-Basic plasmid (Promega, Wisconsin, America). The mutant constructs were generated using the QuickChange II XL site-directed mutagenesis kit (Stratagene, California, America). The firefly luciferase gene was amplified and sub-cloned into the pSin vector to generate the luciferase plasmid. The sequences of shRNAs, primers for cloning and qRT-PCR are listed in Additional file 1: Table S1. Transfection was carried out using Lipofectamine 3000 (Invitrogen, California, America).

### Lentivirus production and transduction

The pSin-luciferase and shRNAs viruses were generated by transfection of the constructs together with pMD2.G and psPAX2 into HEK-293 T cells using calcium phosphate. The medium was replaced with pre-heated fresh DMEM medium 14 h later. The virus particles were harvested twice every 24 h, and filtered by a 0.45 μm filter unit (Millipore, Massachusetts, America). Cells were transduced with recombinant lentivirus with 10 μg/ml polybrene for 48 h and then selected by puromycin for one week.

### Mammosphere culture

5000 cells were seeded into each well of 6-well plates pre-coated with poly (2-hydroxyethyl metacrylate) (Polyhema; Sigma). Cells were cultured in Dulbecco’s modified Eagle's medium (DMEM)/F12 (Gibco) supplemented with B27 (1:50; Gibco), bovine serum albumin (0.4%; Biofroxx), EGF (20 ng/ml; PeproTech), bFGF (20 ng/ml; PeproTech), insulin (5 μg/ml; Sigma), penicillin–streptomycin (Sangon Biotech, Shanghai, China), L-glutamine (Gibco) for about 8 days to allow the generation of mammospheres.

### ALDEFLUOR assay

The cells were stained by using the ALDEFLUOR™ Kit (Stem Cell, Canada) according to the manufacturer’s instructions. In brief, cells were suspended in the assay buffer with 1 × 10^6^ cells/ml. Cells were incubated with ALDH substrate at 37 °C for 30 min, the control group was incubated with ALDH substrate and DEAB. Then, cells were centrifuged to discard the supernatant, re-suspended with assay buffer and analyzed by FACS.

### Conditioned medium and co-culture system

For FACS-sorted BCSCs, cells were stained by ALDEFLUOR™ Kit and suspended in the assay buffer with 5 × 10^6^ cells/ml. The ALDH-positive BCSCs were isolated by flow cytometry sorting. For mammosphere enriched BCSCs, the suspending mammosphere cultured cells were centrifuged at 400 g for 3 min to collect the pellets, and trypsinized to single cells. To harvest the BCSC-derived CM, BCSCs isolated from flow cytometry sorting or mammosphere culturing were seeded in 10 cm dish with regular medium supplemented with 10% FBS. The cells were cultured in monolayer for 48 h to harvest the supernatant conditioned medium. The CM from parallel cultured parental cells was used as control. The CM was collected and centrifuged at 2000 g for 3 min and filtered with a 0.22 μm filter unit (Millipore) to deplete any cell debris. In the Boyden co-culture system (3 μm pore filters; Corning), BCSCs were seeded on the upper chamber, and the same number of parental cells were seeded on the lower compartment. In the control setting, the same number of parental cells were used in both chambers. Cell number in the lower compartment was counted 48 h later. All the cells were cultured with the CM from or co-cultured with the respective same cell line.

### In vitro* migration and invasion assay*

For transwell migration and invasion assays, cells were starved in serum-free medium for 12 h. For migration assays, 1 × 10^5^ pre-starved cells suspended in serum-free medium with 0.2% BSA were plated in the top chamber with non-coated membrane (24-well insert; 8 μm pore size; BD Biosciences). For invasion assay, 1 × 10^5^ pre-starved cells suspended in serum-free medium with 0.2% BSA were plated in the top chamber pre-coated with 12% Matrigel (24-well insert; 8 μm pore size; BD Biosciences). In both assays, medium supplemented with 10% serum was used as a chemoattractant in the lower chamber. The cells were incubated for 8 to 72 h (depending on the cell mobility) and cells that did not migrate or invade through the pores were removed by a cotton swab. The top chambers were fixed with 90% ethanol, stained by 0.1% crystal violet, photographed and cells numbers were counted.

### qRT-PCR and western blot

Total RNA was isolated using TRIzol (Invitrogen). RNA was then converted to cDNA using the RevertAid first strand cDNA synthesis kit (Thermo Scientific, Massachusetts, America). The SYBR Premix Ex Taq kit (Takara, Japan) was used to determine the expression levels, GAPDH served as an input control. Protein was extracted from cells using RIPA lysis buffer. For nuclear β-CATENIN detection, the cytoplasmic and nuclear protein were isolated by the Nuclear Protein and Cytoplasmic Protein Extraction Kit (Beyotime, P0027) according to the manufacturer’s instructions. The antibodies used were listed in Additional file 1: Table S2.

### Luciferase reporter assay

MCF-7 cells were seeded at 60% confluence in 24-well plates. For the ERE4 reporter assay, 0.2 μg ERE4 luciferase reporter plasmid was transfected into cells using lipofectamine 3000 (Invitrogen). For the *IL8* promoter reporter, 0.2 μg pGL3 Basic luciferase reporter was transfected into MCF-7 cells stably transfected with β-CATENIN shRNA or vector. pRL-TK plasmid was provided as an internal transfection control. The transfected cells were lysed 48 h later, and the luciferase activities were determined by the Dual-Luciferase® Reporter Assay System (Promega).

### ChIP assay

Chromatin immunoprecipitation was performed using the ChIP Assay kit (Beyotime, China) and carried out following the manufacturer's instructions. DNA enrichment was assessed by PCR using PrimeStar HS DNA Polymerase (Takara, Japan).

### Immunohistochemistry

Formalin-fixed, paraffin-embedded tissue was cut into 5 μm section, de-paraffinized in xylene, rehydrated through graded ethanol, quenched for endogenous peroxidase activity in 3% (v/v) hydrogen peroxide, and processed for antigen retrieval by heating in 10 mM citrate buffer (pH 6.0) at 100 °C. Sections were incubated at 4 °C overnight with indicated antibodies. Immunostaining was performed using UltraSensitive S-P Detection Kit (KIT-9720, Maixin, China), and then color was developed by using a DAB kit (DAB-0031, Maixin, China). Subsequently, sections were counterstained with hematoxylin. TUNEL assay was performed with an in situ cell death detection kit (Roche) according to the manufacturer’s instructions.

### Statistical analysis

Data were presented as mean ± SD (standard deviation), and GraphPad Prism (San Diego, CA) was used for the statistical analysis. The methods to determine statistical significance in each result are mentioned in the figure legend. All experiments were repeated at least three times. *p* < 0.05 was considered as statistically significant.

## Results

### *BCSCs confer tamoxifen resistance in ER* + *breast cancer cells*

**Fig. 1:**
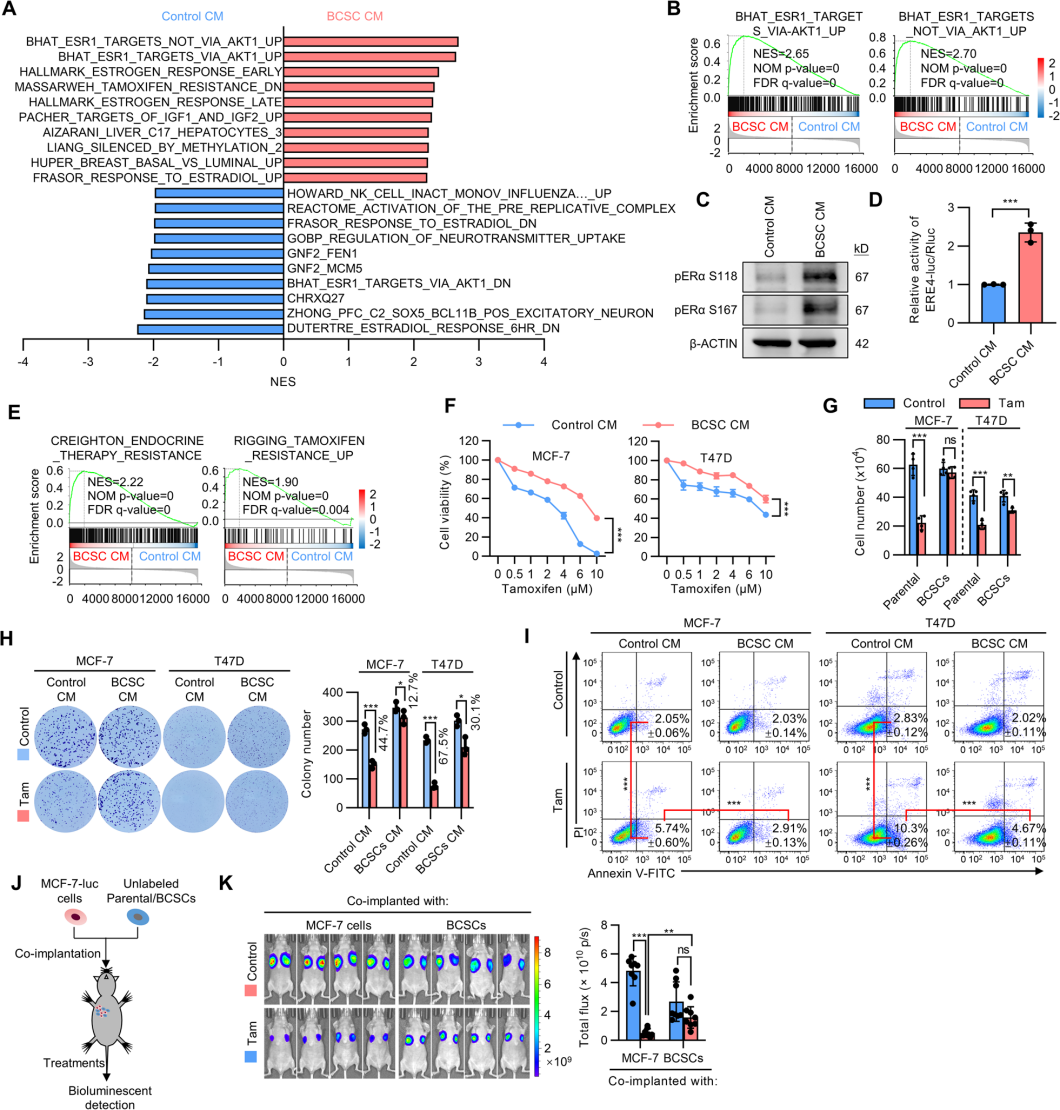
BCSC secretome confers tamoxifen resistance. **A** Top 10 enriched signature gene sets representing RNA-seq data from MCF-7 cells cultured with control CM or BCSC CM. **B** Enrichment of upregulated ESR1 target genes upon culture of MCF-7 cells with BCSC CM by GSEA. **C** Immunoblot showing the phosphorylation of ERα in MCF-7 cells with BCSC CM. **D** The ERα transcriptional activity of estrogen response element (ERE4) in MCF-7 cells with BCSC CM. **E** Enrichment of upregulated genes involved in endocrine therapy resistance or tamoxifen resistance upon culture of MCF-7 cells with BCSC CM by GSEA. **F** MCF-7 or T47D cells were cultured in CM derived from respective mammosphere-enriched BCSCs or parental cells and treated with a graded concentration of tamoxifen for 5 days. Cell viability was determined by MTT assay. **G** MCF-7 or T47D cells were co-cultured with the respective mammosphere-enriched BCSCs or parental cells and treated with 5 μM tamoxifen or vehicle. Cell viability was determined by cell count assay. (H-I) Colony formation assay **H** or Apoptosis (Annexin V-FITC + /PI-) **I** of MCF-7 or T47D cells cultured with CMs derived from respective mammosphere-enriched BCSCs or parental cells and treated with 5 μM tamoxifen or vehicle. **J** The schematic of co-implantation model. 2 × 10^6^ luciferase-labeled MCF-7 cells were co-implanted with 4 × 10^5^ unlabeled parental cells or mammosphere-enriched BCSCs into the second fat pad of host mice. A slow-release tamoxifen pellet was implanted or repertaxin was injected to the treatment group as indicated. **K** In vivo bioluminescent imaging was performed of the tumor burden generated by MCF-7-luc cells. All experiments were repeated at least three times. Results are shown as mean ± S.D. *P < 0.05; **P < 0.01; ***P < 0.001; ns, not significant (Two-way ANOVA test in **F**, others one-way ANOVA followed by Tukey’s multiple comparison)

To characterize the effect of BCSCs on cancer progression, BCSCs enriched by mammosphere culture [19, 20] were cultured in monolayer culture for 48 h to harvest the supernatant of conditioned medium (CM). Unbiased RNA sequencing by use of MCF-7 cells cultured with CM from BCSCs or parental cells was performed (Additional file 2: Fig. S1A). Gene set enrichment analysis (GSEA) suggested that the gene expression pattern of cells cultured with BCSC CM positively correlated with gene signatures of increased estrogen receptor (ER) activity (Fig. 1A), with ERα target genes being potentiated upon culture with BCSC CM (Fig. 1B). Concordantly, culture of MCF-7 cells with BCSC CM increased ERα phosphorylation at both the S118 and S167 sites [21] (Fig. 1C), and markedly increased the transcriptional activity of ERα as determined by use of an estrogen response element (ERE4) reporter assay (Fig. 1D). Furthermore, endocrine therapy resistance and tamoxifen resistance associated genes were enriched in MCF-7 cells cultured with BCSC CM (Fig. 1E). Hence, the possible modulation of the sensitivity to anti-estrogen therapy of ER-positive breast cancer cells was next examined. The ALDEFLUOR assay was used to separate the BCSCs and differentiated cancer cell populations [22]. ER + MCF-7 and T47D cells cultured with CM from either mammospheres or ALDH + cells developed persistent resistance to increasing concentrations of tamoxifen compared to cells cultured in control medium (Fig. 1F and Additional file 2: Fig. S1B). As a control, we also determined if the BCSC secretome could affect chemotherapeutic efficacy in breast cancer. MCF-7 or T47D cells were cultured with CM derived from BCSCs or parental cells and subsequently treated with graded concentrations of doxorubicin, docetaxel or 5-FU. Interestingly, the BCSC secretome did not seem to affect the sensitivity of MCF-7 or T47D cells to the tested cytotoxic drugs (Additional file 2: Fig. S1C, D). A transwell co-culture system was employed for further characterization. Tamoxifen treatment did not alter MCF-7 cell number and only modestly decreased T47D cell number when co-cultured with the respective mammosphere-enriched BCSCs compared to the control cells (Fig. 1G). Similarly, colony formation assay revealed that cells cultured with BCSC-derived CM were either totally resistant to tamoxifen (MCF-7) or exhibited a modest response to tamoxifen (T47D), compared to the distinct sensitivity in the control settings (Fig. 1H). Concordantly, BCSC-derived CM diminished apoptosis in response to tamoxifen in both cell lines compared to the respective control CM as determined by Annexin V/PI staining (Fig. 1I). For in vivo verification*,* 1 × 10^6^ luciferase labeled MCF-7-luc cells were orthotopically co-implanted with 4 × 10^5^ unlabelled mammosphere-enriched BCSCs or parental cells with slow-release E2 pellets. When the xenograft reached 100 mm^3^, slow-release tamoxifen pellets were implanted. The growth of xenografts specially derived from MCF-7-luc cells were determined by bioluminescent imaging (Fig. 1J). It was observed that tamoxifen effectively abrogated the growth of xenografts derived from MCF-7-luc cells co-implanted with parental MCF-7 cells, whereas the xenografts derived from MCF-7-luc cells co-implanted with BCSCs were minimally affected (Fig. 1K), suggesting that BCSCs potently promoted tamoxifen resistance in vivo. Thus, these results demonstrate that BCSCs conferred tamoxifen resistance in ER + breast cancer cells.

### BCSCs enhance EMT and metastatic behavior of breast cancer cells

**Fig. 2:**
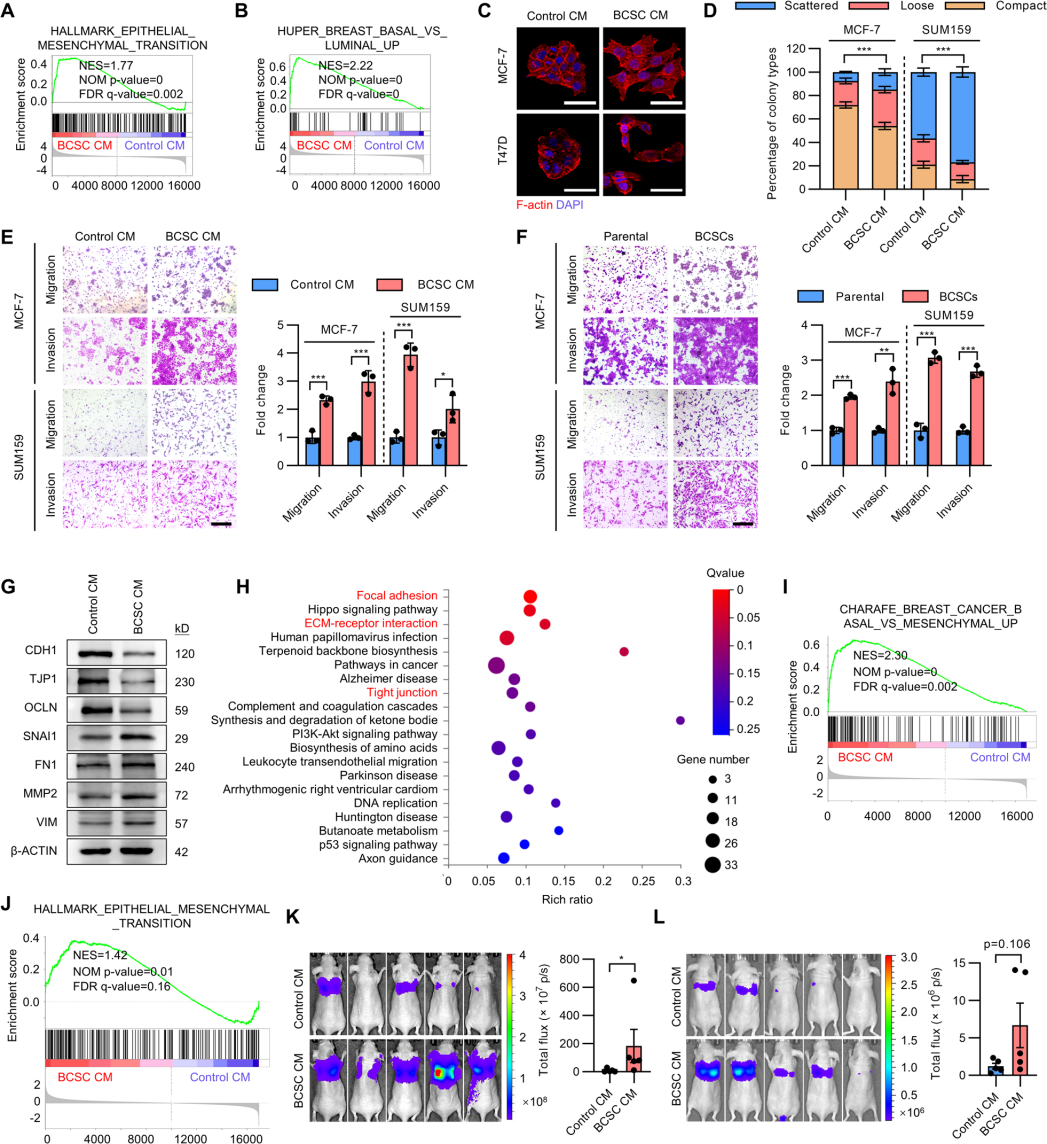
BCSC secretome confers metastasis. **A**, **B** GSEA analysis of EMT associated gene signature **A** or basal-like associated gene signature **B** in MCF-7 cells cultured with BCSC CM. **C** Cell morphology of and F-Actin distribution in MCF-7 and T47D cells cultured with CM from parental cells or BCSCs. **D**, **E** Colony-scattering assays **D** or transwell migration and invasion assay **E** of MCF-7 and SUM159 cells cultured with CM from parental cells or BCSCs. Scale bar: 300 μm. **F** Transwell migration and invasion assay of MCF-7 and SUM159 cells co-cultured with the respective parental cells or BCSCs. Scale bar: 300 μm. **G** Immunoblot of EMT markers in MCF-7 cells cultured with CM from parental cells or BCSCs. **H** KEGG analysis of pathways enriched in MDA-MB-231 cells cultured with BCSC CM compared to that with control CM. **I**, **J** GSEA analysis of the respective gene set enriched in MDA-MB-231 cells cultured with BCSC CM. **K** The MDA-MB-231 cells were cultured with CM from parental cells or BCSCs for 48 h and then injected into the tail vein of nude mice. In vivo bioluminescent imaging was performed of the metastatic burden 2 weeks later. **L** The MDA-MB-231 cells were cultured with CM from parental cells or BCSCs for 48 h and orthotopically injected into the second mammary fat pad of nude mice. The primary tumors were excised 2 weeks later and in vivo bioluminescent imaging was performed of the metastatic burden 4 weeks later. All experiments were repeated at least three times. Results are shown as mean ± S.D. *P < 0.05; **P < 0.01; ***P < 0.001; ns, not significant (unpaired two-tailed Student’s t-test)

Further GSEA analysis demonstrated that epithelial-to-mesenchymal transition (EMT) associated genes were enriched in cells cultured with BCSC CM (Fig. 2A). Additionally, the gene expression pattern of cells cultured with BCSC CM positively correlated with a basal-like gene signature (Fig. 2B). The possible modulation of invasion and metastasis of breast cancer cells by BCSCs was subsequently examined. Indeed, culture of ER + MCF-7 and T47D cells in BCSC CM resulted in a phenotypic transition to a more mesenchymal phenotype, characterized by relatively loose cell colonies and a more elongated cellular morphology (Fig. 2C). In colony-scattering assays, culture of MCF-7 and triple-negative breast cancer (TNBC) SUM159 cells in BCSC CM significantly increased the proportion of scattered and loose colonies while decreased that of compact colonies (Fig. 2D). Furthermore, culturing with BCSC CM significantly increased the cell migration and invasion capacity of MCF-7 and SUM159 cells as shown by use of transwell migration and invasion assays (Fig. 2E) and wound healing assay (Additional file 2: Fig. S2A). Consistently, co-culture with the respective mammosphere-enriched BCSCs also remarkably increased the cell migration and invasion capacity of MCF-7 and SUM159 cells (Fig. 2F). As EMT is a common process that has been postulated to be responsible for carcinoma cell metastasis, the effect of the BCSC secretome on the expression of epithelial markers (CDH1, TJP1 and OCLN) and mesenchymal markers (Snail, FN1, MMP2 and VIM) was assessed. Strikingly, culture with BCSC CM significantly decreased epithelial gene expression and increased mesenchymal gene expression in MCF-7 cells (Fig. 2G). The highly metastatic TNBC MDA-MB-231 cells also exhibited increased migration and invasion upon culture with BCSC CM (Additional file 2: Fig. S2B). Unbiased RNA sequencing by use of MDA-MB-231 cells cultured with CM from BCSCs or parental cells was further performed. KEGG pathway enrichment analysis suggested that the focal adhesion, ECM-receptor interaction and tight junction pathways were enriched in cells cultured with BCSC CM (Fig. 2H). Concordantly, GO analysis also suggested that the focal adhesion, adherens junction and cell junction associated genes were enriched in cells cultured with BCSC CM (Additional file 2: Fig. S2C), indicative that the adhesive and migrative capacity were potentially increased in MDA-MB-231 cells cultured with BCSC CM. GSEA analysis further suggested that the basal-like and EMT associated genes were enriched in MDA-MB-231 cells cultured with BCSC CM (Fig. 2I, J). For in vivo assessment, MDA-MB-231 cells were cultured with CM from BCSCs or parental cells for 48 h and then injected into the tail vein of nude mice. Lung metastasis was determined by bioluminescent imaging. Consistently, an increased metastatic burden was observed in mice injected with MDA-MB-231 cells pre-cultured with BCSC CM compared to that pre-cultured with control CM (Fig. 2K and Additional file 2: Fig. S2D). Additionally, MDA-MB-231 cells pre-cultured with BCSC CM or control CM were orthotopically injected into the second mammary fat pad of nude mice. The primary tumors were excised 2 weeks later and the metastatic burden was assessed 4 weeks after implantation. Although not statistically significant, culturing with BCSC CM considerably increased the spontaneous metastatic capacity of MDA-MB-231 cells. (Fig. 2L). Hence, these data indicate that the BCSC secretome endows invasive and metastatic properties to breast cancer cells in vitro and in vivo.

### BCSCs-derived IL8 drives tamoxifen resistance

**Fig. 3:**
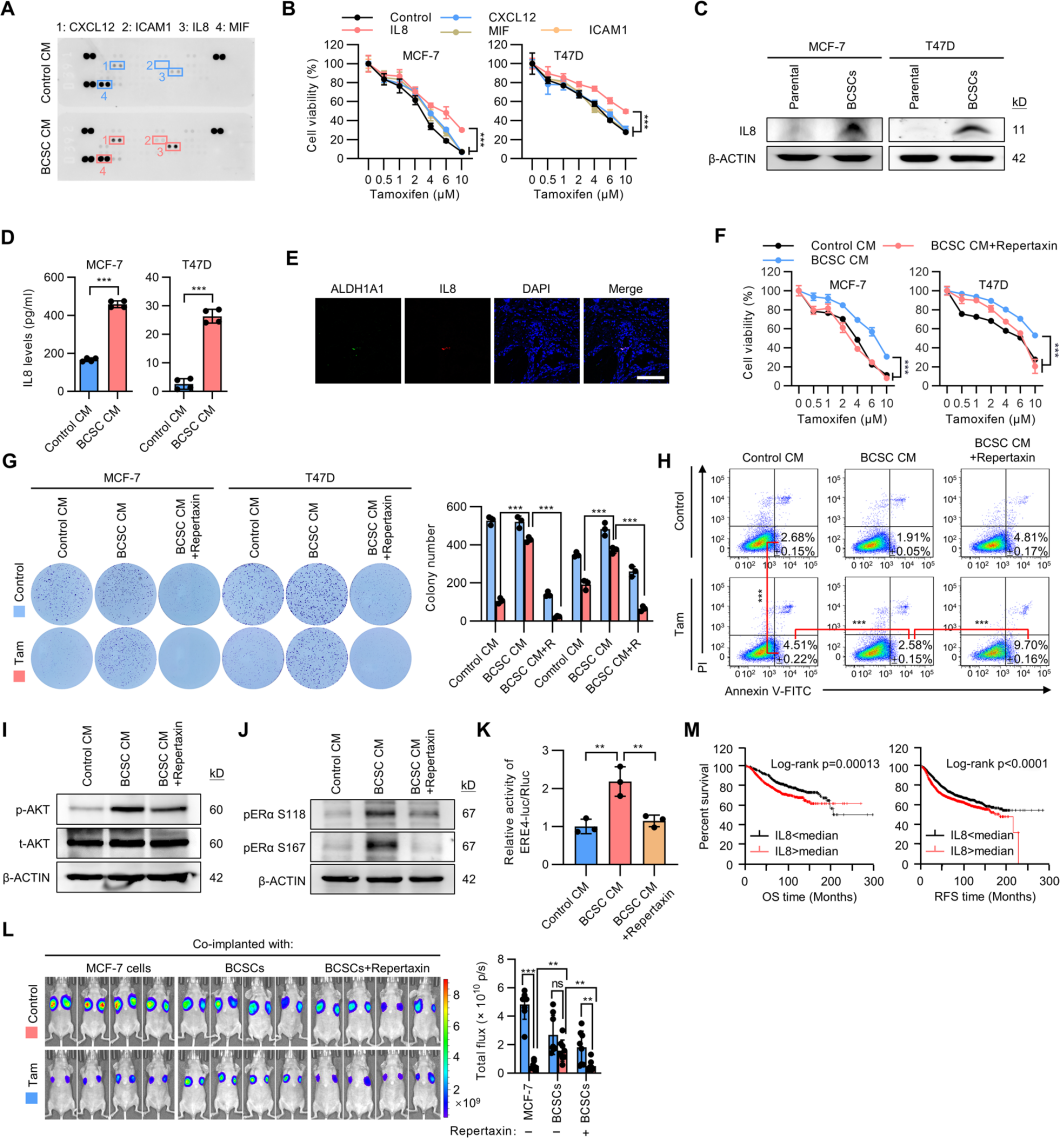
BCSC-derived IL8 drives tamoxifen resistance. **A** Protein array analysis of factors present in CM derived from mammosphere-enriched BCSCs or parental MCF-7 cells. **B** MCF-7 and T47D cells were cultured in the presence of the indicated recombinant proteins (100 ng/ml) and treated with a graded concentration of tamoxifen for 5 days. Cell viability was determined by MTT assay. **C** Immunoblot analysis of IL8 protein levels in matched mammosphere-enriched BCSCs and parental MCF-7 or T47D cells. **D** ELISA quantification of secreted IL8 levels in CM from paired mammosphere-enriched BCSCs and parental MCF-7 or T47D cells. **E** Immunofluorescence staining of ALDH1A1 and IL8 in breast cancer tissues. Scale bar: 100 μm. **F** MCF-7 or T47D cells were cultured with CM derived from respective parental cells or mammosphere-enriched BCSCs in the presence or absence of 0.1 mM repertaxin and treated with a graded concentration of tamoxifen for 5 days. Cell viability was determined by MTT assay. **G**, **H** Colony formation assay **G** or Apoptosis (Annexin V-FITC + /PI-) **H** of MCF-7 or T47D cells cultured with the CM derived from respective parental cells or mammosphere-enriched BCSCs in the presence or absence of 0.1 mM repertaxin and treated with 5 μM tamoxifen. **I** Immunoblot showing the activity of the AKT (p-AKT) pathways in MCF-7 cells cultured with the CM derived from parental cells or mammosphere-enriched BCSCs in the presence or absence of 0.1 mM repertaxin. **J**, **K** Immunoblot showing the phosphorylation of ERα **J** or the ERα transcriptional activity of estrogen response element (ERE4) **K** in MCF-7 cells cultured with the CM derived from parental cells or mammosphere-enriched BCSCs in the presence or absence of 0.1 mM repertaxin. **L** The MCF-7 and BCSCs group are same as Fig. 1K. A rescue group with Repertaxin treatment was included, and i*n vivo* bioluminescent imaging was performed of the tumor burden generated by MCF-7-luc cells. **M** Kaplan–Meier plots of overall survival (OS) or relapse-free survival (RFS) in breast cancer patients stratified according to their *IL8* levels. The data was analyzed by Kaplan–Meier plotter software. All experiments were repeated at least three times. Results are shown as mean ± S.D. *P < 0.05; **P < 0.01; ***P < 0.001; ns, not significant (Unpaired two-tailed Student’s t-test in **D**, one-way ANOVA followed by Tukey’s multiple comparison in **H** and 3 K-3L, others Two-way ANOVA test)

The mechanism by which BCSCs endowed tamoxifen resistance and metastasis to breast cancer cells was next determined. A cytokine antibody array was employed to screen for differentially secreted cytokines in CM from BCSCs and parental MCF-7 cells. CXCL12, ICAM1, IL8, and MIF were among the prominent cytokines with elevated levels in the CM from BCSCs (Fig. 3A and Additional file 2: Fig. S3A). Further functional characterization by supplementation of the respective recombinant proteins (CXCL12, ICAM1, IL8, and MIF) identified IL8 as the only protein which rendered significant tamoxifen resistance in both MCF-7 and T47D cells (Fig. 3B). The mRNA level and cytoplasmic/secreted protein levels of IL8 were verified to be consistently much higher in BCSCs compared to parental cells (Additional file 2: Fig. S3B, C and Fig. 3C, D). ALDH1 is a marker of normal and malignant human mammary stem cells [22]. Co-immunofluorescent analysis in ER-positive breast cancer specimens further verified that IL8 preferentially marked ALDH1-positive cancer cells, indicating the exclusive expression of IL8 in BCSCs (Fig. 3E). CXCR1 and CXCR2, the cognate receptors of IL8, were shown to be expressed in both parental cells and BCSCs (Additional file 2: Fig. S3D). Supplementation of recombinant IL8 did not affect the proportion of BCSCs in MCF-7 and T47D cells (Additional file 2: Fig. S3E). Thus, BCSC-derived IL8 mediates tamoxifen resistance of the bulk breast cancer cell population.

We next determined if targeting IL8 derived from BCSCs would ameliorate tamoxifen resistance. As expected, repertaxin, a small molecule inhibitor of IL8, which specifically binds to IL8 receptors CXCR1/2, significantly attenuated tamoxifen resistance in MCF-7 and T47D cells cultured with BCSC-derived CM (Fig. 3F). Similar findings were observed using an IL8 neutralizing antibody (Additional file 2: Fig. S3F). Additionally, BCSC CM derived from IL8 depleted cells failed to improve tamoxifen resistance compare to that derived from control cells (Additional file 2: Fig. S3G–H). Colony formation assay revealed that while cells cultured with BCSC-derived CM were resistant to tamoxifen treatment, this effect was reversed by supplementation with repertaxin (Fig. 3G). Repertaxin also dramatically abrogated the decreased apoptosis observed upon tamoxifen treatment of cells cultured in BCSC CM (Fig. 3H). The AKT signaling pathway has been reported to be activated by IL8, and required for tamoxifen resistance in breast cancer [23, 24]. Consistently, repertaxin diminished the phosphorylation of AKT induced by BCSC CM (Fig. 3I). It has been well established that ERα can be phosphorylated by AKT signaling in a ligand-independent manner [25]. Importantly, inhibition of IL8 signaling by repertaxin attenuated ERα phosphorylation induced by the BCSC secretome (Fig. 3J). Additionally, repertaxin also reversed increased ERE reporter activity observed in cells cultured with BCSC CM (Fig. 3K). The involvement of AKT signaling in decreased tamoxifen responsiveness mediated by BCSC secretome was further investigated. As expected, treatment with an AKT inhibitor also significantly attenuated tamoxifen resistance in MCF-7 cells cultured with BCSC-derived CM (Additional file 2: Fig. S3I). In vivo, repertaxin attenuated BCSC promoted tamoxifen resistance in the accompanying MCF-7-luc cells (Fig. 3L). We further analyzed the correlation of IL8 with the survival outcomes of breast cancer patients in the published datasets. Consistently, in breast cancer patients, higher expression of *IL8* in the primary cancer was correlated with poor overall survival and relapse-free survival outcomes (Fig. 3M). Further analysis suggested that higher *IL8* level was correlated with poor survival outcomes in both ER-positive and TNBC breast cancer patients (Additional file 2: Fig. S3J). Collectively, these results indicated that BCSC-secreted IL8 promotes tamoxifen resistance of ER + breast cancer cells.

### Targeting IL8 reverses tamoxifen resistance and increases sensitivity to fulvestrant and palbociclib

**Fig. 4:**
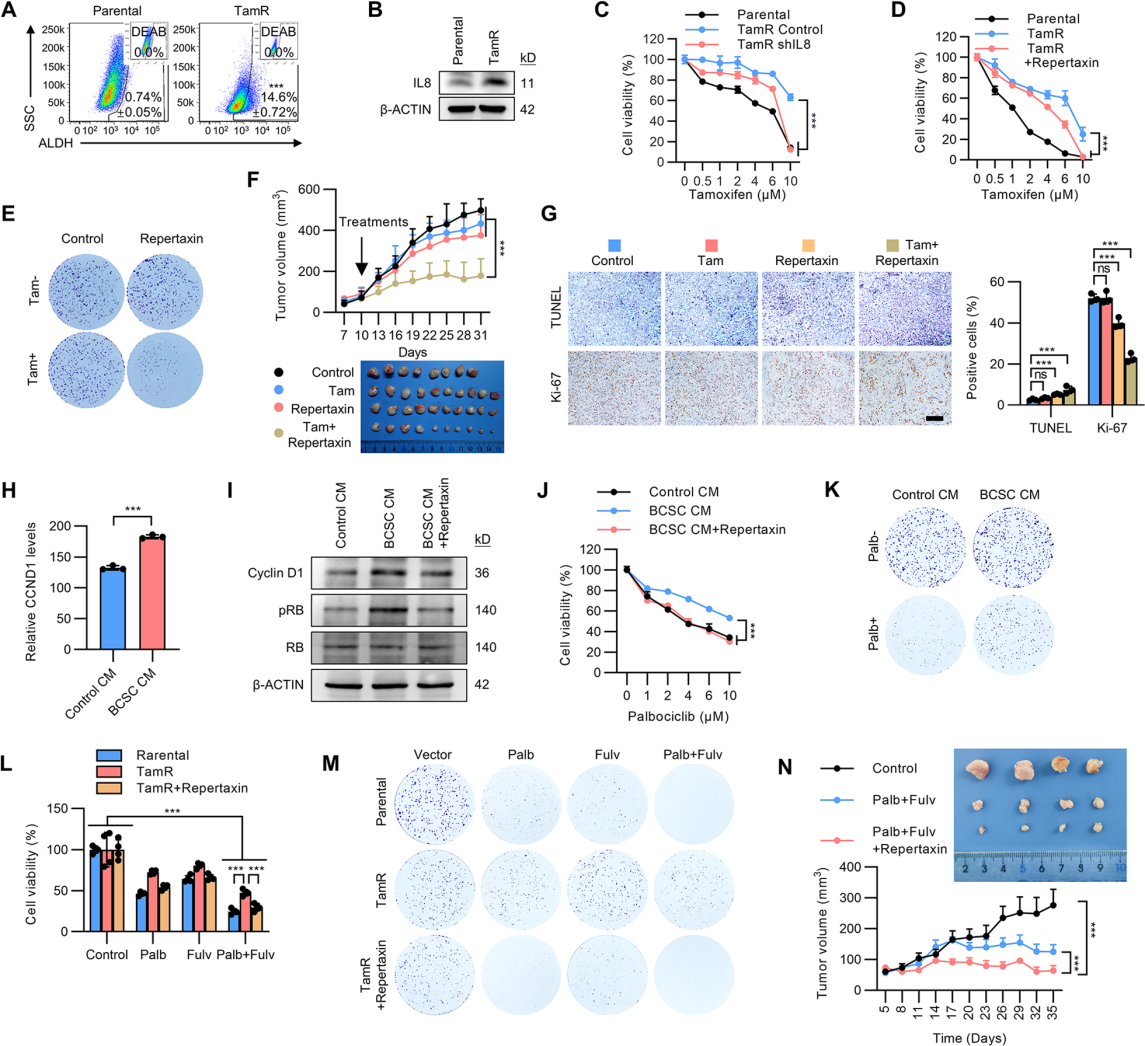
Targeting IL8 overcomes tamoxifen resistance. **A** FACS analysis of the proportion of ALDH + BCSCs in the matched MCF-7 cells and tamoxifen-resistant MCF-7 TamR cells. **B** Immunoblot analysis of IL8 levels in the matched MCF-7 cells and tamoxifen-resistant MCF-7 TamR cells. **C** The matched MCF-7 or MCF-7 TamR cells transfected with vector or IL8 shRNA were treated with a graded concentration of tamoxifen for 5 days. Cell viability was determined by MTT assay. **D** The matched MCF-7 or MCF-7 TamR cells were treated with a graded concentration of tamoxifen for 5 days in the presence or absence of 0.1 mM repertaxin. Cell viability was determined by MTT assay. **E** Colony formation assay of MCF-7 or MCF-7 TamR cells treated with treated with 5 μM tamoxifen and in the presence or absence of 0.1 mM repertaxin. **F** The growth curves (left) or image (right) of tumors derived from MCF-7 TamR cells for 30 days. The mice were treated with slow-release tamoxifen supplement alone, repertaxin alone, or combined tamoxifen and repertaxin. **G** The representative image (left) and statistical result (right) of Ki-67 staining and TUNEL labeling of the respective MCF-7 TamR cells-derived tumor sections as indicated. Scale Bar: 100 μm. **H** RNA-sequencing analysis of the CCND1 levels in MCF-7 cells cultured with CM fom BCSCs or parental cells. **I** Immunoblot analysis of Cyclin D1, pRb and RB levels in MCF-7 cells cultured with the indicated CM. **J** MCF-7 cells were cultured with CM derived from parental cells or BCSCs in the presence or absence of 0.1 mM repertaxin and treated with a graded concentration of palbociclib. Cell viability was determined by MTT assay. **K** Colony formation assay MCF-7 cells cultured with control CM or BCSC CM in the presence or absence of palbociclib. **L**, **M** MTT **L** or colony formation assay **M** of MCF-7 parental, TamR cells in the presence or absence of 0.1 mM repertaxin and treated with 1 μM fulvestrant and/or palbociclib. **N** Tumor growth curve of MCF-7 TamR cells treated with fulvestrant/ palbociclib and/or repertaxin. All experiments were repeated at least three times. Results are shown as mean ± S.D. *P < 0.05; **P < 0.01; ***P < 0.001; ns, not significant (Unpaired two-tailed Student’s t-test in **A, H**, one-way ANOVA followed by Tukey’s multiple comparison in **G, L**, others Two-way ANOVA test)

It was next determined if targeting IL8 could overcome acquired tamoxifen resistance. Interestingly, the percentage of ALDH + cells and IL8 levels were increased in an acquired tamoxifen resistance model (MCF-7 TamR cells) [26] (Fig. 4A, B). IL8 depletion by shRNA re-sensitized MCF-7 TamR cells to tamoxifen treatment (Fig. 4C), whereas depletion of MIF, CXCL12 or ICAM1 in MCF-7 TamR cells did not affect tamoxifen sensitivity (Additional file 2: Fig. S4A, B). Blocking IL8 signaling by using repertaxin also significantly re-sensitized MCF-7 TamR cells to tamoxifen treatment (Fig. 4D). A similar result was observed in colony formation assay (Fig. 4E and Additional file 2: Fig. S4C). Given that the AKT signaling pathway was activated by IL8 [23], treatment with AKT inhibitor in MCF-7 TamR cells also significantly reversed the acquired tamoxifen resistance (Additional file 2: Fig. S4D). The therapeutic potential of pharmacological inhibition of IL8 in tamoxifen-resistant breast cancer was further evaluated. Repertaxin has been utilized in phase 2 clinical trials for organ transplant (NCT01220856 and NCT00224406), indicating tolerability for in vivo application. Xenografts derived from TamR cells were insensitive to tamoxifen treatment as expected and repertaxin alone only slightly inhibited the growth of TamR xenografts (Fig. 4F). Strikingly, combined treatment with tamoxifen and repertaxin led to a 70% reduction of xenograft volume as compared to the control or tamoxifen treatment alone (Fig. 4F). IHC analyses of xenograft sections showed that combined application of tamoxifen and repertaxin increased the percentage of apoptotic cells and decreased the percentage of proliferative cells in TamR xenografts (Fig. 4G). In addition, repertaxin treatment decreased phosphorylated AKT levels in the TamR xenografts (Additional file 2: Fig. S4E). Hence, pharmacological targeting of IL8 may constitute a potential approach to overcome tamoxifen resistance.

According to NCCN Clinical Practice Guidelines 2021 for breast cancer, combined or single use of fulvestrant and/or CDK4/6 inhibitors have been recommended as the first-line or second-line therapeutic regimen for patients with tamoxifen treatment failure [27]. The expression level of Cyclin D1, an ER responsive gene, was indicated for CDK4/6 inhibitor treatment response [28, 29]. As ER signaling was potentiated by the BCSC secretome, it was further investigated if the BCSC secretome modulates the expression of CYCLIN D1 as well as the sensitivity to CDK4/6 inhibitor in breast cancer. Analysis of the RNA-sequencing data revealed that the mRNA level of CCND1 was significantly elevated in MCF-7 cells cultured with BCSC CM (Fig. 4H). Consistently, whereas the protein level of Cyclin D1 was upregulated in MCF-7 cells cultured with BCSC CM, this effect was partially abolished by repertaxin (Fig. 4I). Cyclin D1 and the CDK4/6 complex phosphorylates retinoblastoma protein (Rb), which plays a pivotal role in cell cycle progression [30]. Similar results were observed with the phosphorylated Rb levels, indicating that BCSC-secreted IL8 regulated the expression of Cyclin D1. We next investigated if BCSC-secreted IL8 modulates the sensitivity of CDK4/6 inhibitor in ER + breast cancer cells. As expected, culture with BCSC CM significantly decreased the sensitivity of palbociclib treatment in MCF-7 cells; this effect was reversed by repertaxin (Fig. 4J, K and Additional file 2: Fig. S4F). Tamoxifen-resistant MCF-7 TamR cells exhibited slight response to fulvestrant or/and palbociclib treatment as compared to parental cells (Fig. 4L, M and Additional file 2: Fig. S4G), combined treatment of repertaxin and fulvestrant or/and palbociclib exhibited a synergetic effect (Fig. 4L, M and Additional file 2: Fig. S4G). For in vivo assessment, MCF-7 TamR cells were orthotopically injected into the mammary fat pad of nude mice supplemented with estrogen. When the xenograft volume reached approximately 100 mm^3^, the mice were treated with combined fulvestrant and palbociclib in the presence or absence of repertaxin treatment. While fulvestrant and palbociclib treatment resulted in a moderate decrease in xenograft growth, combined repertaxin, fulvestrant and palbociclib treatment dramatically reduced the xenograft volume (Fig. 4N). Ki-67 and TUNEL staining indicated that proliferation was decreased and apoptotic cells were increased in the repertaxin, fulvestrant and palbociclib combined treatment group (Additional file 2: Fig. S4H, I). These data collectively suggest that targeting IL8 facilitates anti-estrogen-resistant breast cancer therapy.

### BCSCs-derived IL8 drives breast cancer metastasis

**Fig. 5:**
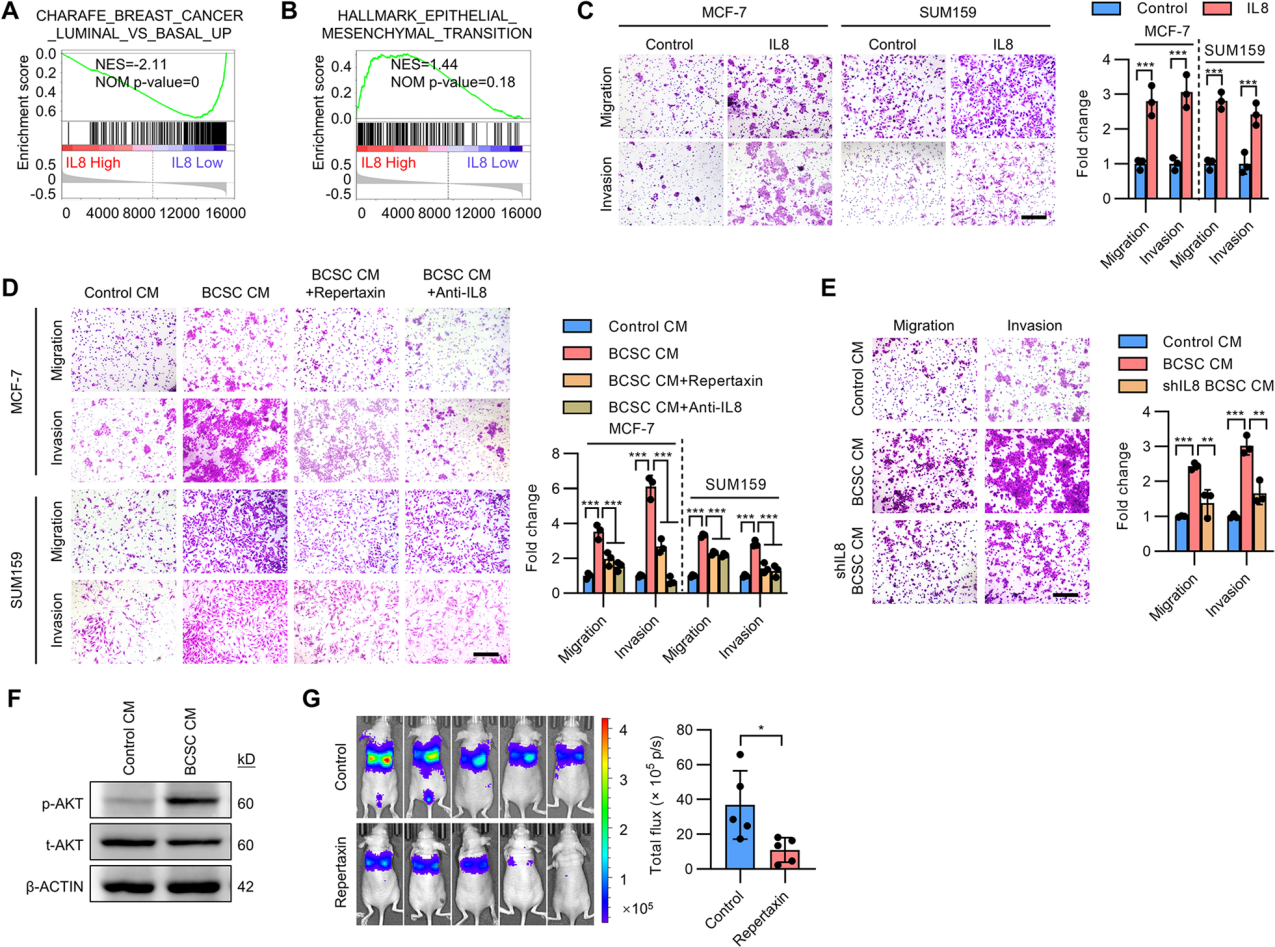
BCSC-derived IL8 drives metastasis. **A**, **B** GSEA analysis of basal-like **A** or EMT associated gene signature **B** or basal-like associated gene signature in TCGA breast cancer patients stratified according to their *IL8* levels. **C** Transwell migration and invasion assay of MCF-7 and SUM159 cells treated with 100 ng/ml recombinant IL8. Scale bar: 300 μm. **D** Transwell migration and invasion assay of MCF-7 and SUM159 cells cultured with CM derived from respective parental cells or mammosphere-enriched BCSCs in the presence or absence of 0.1 mM repertaxin or 1 μg/ml IL8 neutralizing antibody. Scale bar: 300 μm. **E** Transwell migration and invasion assay of MCF-7 and SUM159 cells cultured with CM derived from parental cells or mammosphere-enriched shCONT-BCSCs or shIL8-BCSCs. Scale bar: 300 μm. **F** Immunoblot analysis of p-AKT and t-AKT in MDA-MB-231 cells cultured with CM from BCSCs or parental cells. **G** MDA-MB-231 cells were tail vein injected into nude mice and received repertaxin treatment for 3 weeks, the metastatic burden was determined by bioluminescent imaging. All experiments were repeated at least three times. Results are shown as mean ± S.D. *P < 0.05; **P < 0.01; ***P < 0.001; ns, not significant (Unpaired two-tailed Student’s t-test)

It was next determined if BCSC-secreted IL8 would also promote dissemination. GSEA analysis of The Cancer Genome Atlas (TCGA) in breast cancer patients suggested that patients with higher *IL8* levels were correlated with a basal-like breast cancer signature (Fig. 5A). The expression levels of *IL8* were significantly higher in triple-negative breast cancer (TNBC) patients compared to luminal BC patients (Additional file 2: Fig. S5A). Additionally, EMT-associated genes were also enriched in patients with higher *IL8* levels (Fig. 5B). *IL8* levels were negatively correlated with epithelial markers while positively correlated with a series of mesenchymal markers in the TCGA breast cancer datasets (Additional file 2: Fig. S5B). Hence, BCSC-derived IL8 might be involved in breast cancer metastasis.

Further functional characterization suggested that recombinant IL8 significantly increased the migrative and invasive capacity of both MCF-7 and SUM159 cells (Fig. 5C). In contrast, IL8 depletion by shRNA in MCF-7 and SUM159 cells reduced the migratory and invasive cells (Additional file 2: Fig. S5C). It was next determined if targeting BCSC-derived IL8 could abrogate metastasis. As expected, treatment with either repertaxin or IL8 neutralizing antibody consistently reversed the invasive properties of both cell lines afforded by BCSC secretome (Fig. 5D). Furthermore, BCSC CM derived from IL8 depleted cells failed to improve the migratory and invasive capacity compared to that derived from control cells (Fig. 5E). Additionally, inhibition of IL8 downstream AKT signaling also abolished the BCSC secretome afforded migratory and invasive capacity (Additional file 2: Fig. S5D). It was next determined if targeting IL8 could reduce metastatic disease burden in vivo. Treatment with repertaxin significantly reduced the migration and invasion of the highly metastatic MDA-MB-231 cells (Additional file 2: Fig. S5E). Mechanistic studies revealed that enhanced AKT activity was observed in MDA-MB-231 cells treated with BCSC CM compared to that with control CM (Fig. 5F). CXCR1 and CXCR2 were also shown to be expressed in both parental cells and BCSCs (Additional file 2: Fig. S5F). Supplementation of recombinant IL8 did not affect the proportion of BCSCs in MDA-MB-231 cell culture (Additional file 2: Fig. S5G), suggesting that BCSC-derived IL8 mediates invasiveness via the bulk cancer cell population. For in vivo studies, nude mice were tail vein injected with MDA-MB-231 cells. After 24 h of injection, mice were treated with repertaxin for 3 weeks and the metastatic burden was determined by bioluminescent imaging. Strikingly, repertaxin treatment led to a 70% reduction of the metastatic burden as compared to the control group (Fig. 5G). H & E staining of the lung sections also suggested that the size of the metastatic foci was decreased upon repertaxin treatment (Additional file 2: Fig. S5H). Thus, targeting BCSC-derived IL8 may overcome the burden of metastatic disease.

### β-CATENIN activity in BCSCs promotes IL8 expression

**Fig. 6:**
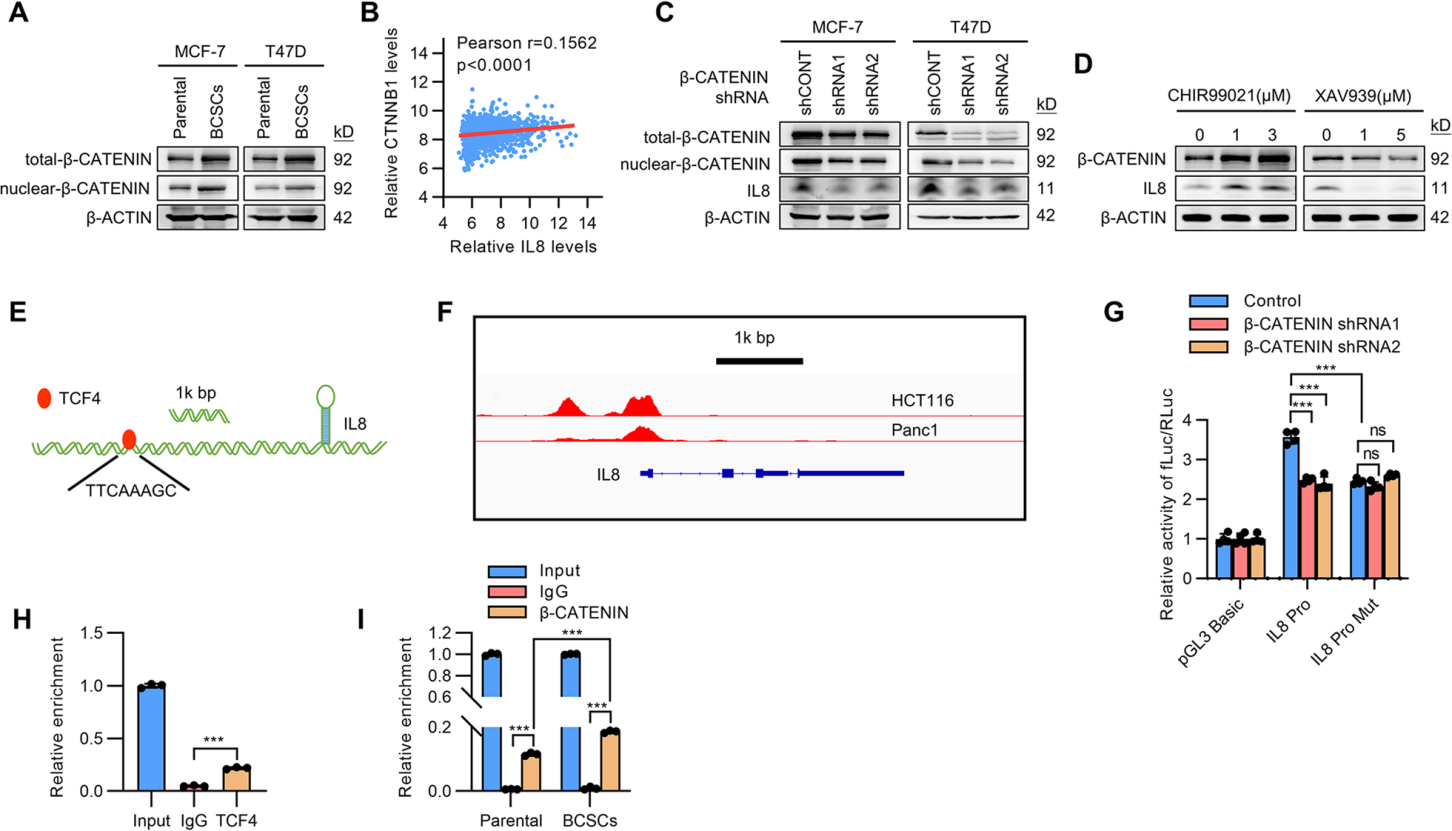
β-CATENIN regulates the transcription of IL8. **A** Immunoblot assessment of the total and nuclear levels of β-CATENIN in the matched mammosphere-enriched BCSCs and parental MCF-7 or T47D cells. **B** The correlation of *CTNNB1* and *IL8* was analyzed in TCGA breast cancer dataset. **C** Immunoblot assessment of the total and nuclear levels of β-CATENIN or IL8 in MCF-7 or T47D cells transfected with two different shRNAs to β-CATENIN. **D** Immunoblot assessment of the protein levels of β-CATENIN or IL8 in MCF-7 cells treated with CHIR99021 or XAV939. **E** Schematic representation of the predicted TCF4 binding sites in the promoter region of *IL8* based on rVista 2.0 software. **F** The ChIP-sequencing data shows the enrichments of TCF4 around the promoter region of IL8 were analyzed in the GEO database (GSM782123 and GSM816437). **G** The regulation of wild-type or TCF4 binding site mutant *IL8* promoter activities by β-CATENIN were determined by luciferase reporter assay. The luciferase reporter activities in the transfected MCF-7 cells were determined with Renilla luciferase activity as input control. **H** ChIP assay analysis of the enrichment of TCF4 to the *IL8* promoter region in MCF-7 cells. **I** ChIP assay analysis of the enrichment of β-CATENIN to the *IL8* promoter region in BCSCs or parental MCF-7 cells. All experiments were repeated at least three times. Results are shown as mean ± S.D. *P < 0.05; **P < 0.01; ***P < 0.001; ns, not significant (One-way ANOVA followed by Tukey’s multiple comparison test)

We next investigated the mechanism underlying the increased expression of IL8 in BCSCs. The β-CATENIN signaling pathway has been reported to play crucial roles in stem cell maintenance and to be elevated in CSCs [31, 32]. It was confirmed that both the total and nuclear β-CATENIN levels were higher in BCSCs than that in parental cells (Fig. 6A). Analysis of the TCGA breast cancer datasets revealed that *IL8* levels were positively correlated with β-CATENIN (*CTNNB1*) levels (Fig. 6B), indicating that the expression of *IL8* might be regulated by β-CATENIN signaling. β-CATENIN depletion in MCF-7 and T47D cells by shRNAs resulted in significantly decreased IL8 levels as well as total and nuclear β-CATENIN levels (Fig. 6C). Consistently, the WNT/β-CATENIN signaling activator, CHIR99021, significantly increased β-CATENIN as well as IL8 levels (Fig. 6D). In contrast, the WNT/β-CATENIN signaling inhibitor, XAV939, diminished the protein levels of β-CATENIN and IL8 (Fig. 6D). BCSC CM derived from β-CATENIN depleted cells failed to endow tamoxifen-resistance and invasiveness to MCF-7 cells (Additional file 2: Fig. S6A, B), supporting the dependence on β-CATENIN signaling for IL8 expression.

It was next determined whether the expression of IL8 is transcriptionally regulated by β-CATENIN signaling. The transcription factor TCF/LEF family has been reported to directly bind to the promoter of the downstream genes of β-CATENIN and activate gene transcription when binding with β-CATENIN [33]. Bioinformatics software rVista 2.0 was employed and predicted one TCF4 binding site in *IL8* promoter (Fig. 6E). Consistently, analysis of the ChIP-sequencing data also revealed the enrichment of TCF4 in the promoter region of *IL8* (Fig. 6F). To study the transcriptional activity of β-CATENIN on the *IL8* promoter, the promoter sequence of IL8 was cloned into the luciferase reporter pGL3 basic plasmid. β-CATENIN depletion in MCF-7 cells decreased the luciferase reporter activity of the wild-type promoter construct (Fig. 6G). In contrast, mutation of the TCF4 binding site in *IL8* promoter abrogated the β-CATENIN responsiveness (Fig. 6G). Further ChIP study confirmed the direct binding of TCF4 on the predicted region of *IL8* promoter (Fig. 6H). ChIP study also revealed increased enrichment of β-CATENIN on the *IL8* promoter in BCSCs compared to that in parental cells (Fig. 6I). Therefore, elevated β-CATENIN signaling in BCSCs transcriptionally activates the expression of *IL8*.

## Discussion

Cancer therapeutic failure may be partly attributed to the poor efficacy of targeting CSCs, which are intrinsically resistant to multiple therapeutic approaches and often substantially enriched following extended drug treatment [34, 35]. However, despite the expansion of the CSC population, non-CSCs remain the majority in the cancer cell population exhibiting drug resistance [11, 34]. How CSCs orchestrate the entire cancer cell population, especially non-CSCs in cancer progression, remains largely unknown.

In this study, we presented evidence that BCSC secretome potentiated ER signaling, leading to reduced sensitivity to tamoxifen and palbociclib in ER + breast cancer cells. Screening of the BCSC secretome identified IL8 as a key factor secreted by BCSCs to promote tamoxifen resistance and reduce palbociclib sensitivity. Although IL8 has been reported to regulate cancer growth, drug resistance and metastasis in various cancers [36–38], the cellular source of IL8 was uncertain. Herein, we reported that IL8 is predominantly expressed in BCSCs; which generated a paradigm by which IL8 afforded paracrine regulation of the bulk cancer cells in the therapeutic response. IL8 significantly enhanced ER activity by cross-talking with key molecules in ER signaling. Consistently, targeting IL8 with Repertaxin not only increased the efficacy of Fulvestrant and/or Palbociclib therapy in ER + breast cancer cells, but also restored the sensitivity to tamoxifen, as well as increased the efficacy of Fulvestrant and/or Palbociclib therapy in tamoxifen resistant cells. Thus, targeting IL8 may provide a novel approach to improve the existing therapeutic regimes for ER + breast cancer patients or those with de novo or acquired resistance to anti-estrogen therapies.

Previous studies suggested a linkage may exist between therapeutic resistance and metastasis [39, 40]. We and others have shown the involvement of CSC in EMT and metastasis [41, 42]. Whether CSCs could orchestrate the entire metastatic process is unclear. Herein, it was further revealed that paracrine secretion of IL8 by BCSCs enabled bulk cancer cells to acquire mesenchymal properties and metastasize. Targeting IL8 with Repertaxin decreased the metastatic burden of highly metastatic MDA-MB-231 cells. Thus, pharmacological inhibition of IL8 may represent a potential prophylactic approach to reduce breast cancer metastatic burden. It should be noted that the microenvironment of the primary tumor endowed invasiveness and anti-apoptotic capacity to the spontaneously metastasizing cancer cells, which may lead to distinct features between spontaneously metastasizing cancer cells and intravenously injected cancer cells [43]. BCSCs also potentiated the spontaneous metastatic capacity of breast cancer cells.

The current literature focus of the CSC secretome has mainly concentrated on two aspects: First, the impact on the components of the tumor microenvironment, such as tumor-associated fibroblasts, macrophages and NK cells [44]; and second, the autocrine regulation of CSCs on self-renewal and differentiation [45]. This study has presented a concept in which CSCs regulate non-CSC therapeutic response and metastasis via paracrine secretion of IL8. Although a series of approaches have been developed to directly target CSCs, toxicity to normal stem cells has limited their clinical application [46]. Under this scenario, it would be expected that targeting IL-8 by using the phase 2 clinical trial utilized Repertaxin may exhibit improved tolerability in the clinical settings.

## Conclusion

This study presented a novel paradigm by which CSCs cooperate with non-CSCs to promote treatment resistance and metastasis. Pharmacological inhibition of IL8 may represent a potential approach to improve existing therapeutic regimes for breast cancer patients with de novo or acquired resistance to anti-estrogen therapies and to prophylactically alleviate metastatic diseases.

## Supplementary Information

**Additional file 1: Table S1**. Sequences of shRNAs, and primers for cloning, ChIP assays, and qRT-PCR analysis. **Table S2**. List of antibodies used in western blot, immunohistochemistry and chromatin immunoprecipitation assay.

**Additional file 2: Fig. S1**. BCSC secretome confers tamoxifen resistance. (A) The schematic of BCSC conditioned medium and RNA-sequencing. (B) T47D cells were cultured in CM derived from ALDEFLUOR assay sorted BCSCs or differentiated cancer cells and treated with a graded concentration of tamoxifen for 5 days. Cell viability was determined by MTT assay. (C-D) MCF-7 (C) or T47D (D) cells were cultured in CM derived from the respective mammosphere-enriched BCSCs or parental cells and treated with a graded concentration of doxorubicin, docetaxel or 5-FU for 5 days. Cell viability was determined by MTT assay. All experiments were repeated at least three times. Results are shown as mean ± S.D. *P<0.05; **P<0.01; ***P<0.001; ns, not significant (Two-way ANOVA test). **Fig. S2**. BCSC secretome confers metastasis. (A) In vitro wound-healing assay MCF-7 and SUM159 cells cultured with CM from parental cells or BCSCs. Scale bar: 300 μm. (B) Transwell migration and invasion assay of MDA-MB-231 cells cultured with CM from parental cells or BCSCs. Scale bar: 300 μm. (C) H&E staining of lung metastasis derived from MDA-MB-231 cells cultured with CM from parental cells or BCSCs. Scale bar: 300 μm. (C) GO analysis of genes enriched in MDA-MB-231 cells cultured with BCSC Cm compared to that with control CM. (D) H&E staining of lung sections derived from tail vein injected MDA-MB-231 cells cultured with control CM or BCSC CM. Scale bar: 300 μm. All experiments were repeated at least three times. Results are shown as mean ± S.D. *P<0.05; **P<0.01; ***P<0.001; ns, not significant (Unpaired two-tailed Student’s t-test). **Fig. S3**. BCSC-derived IL8 drives tamoxifen resistance. (A) Densitometric analysis of the protein array of factors present in CM derived from mammosphere-enriched BCSCs or parental MCF-7 cells. (B) qRT-PCR quantification of IL8 mRNA levels in matched mammosphere-enriched BCSCs and parental MCF-7 or T47D cells. (C) ELISA quantification of secreted IL8 protein levels in CM from paired ALDEFLUOR assay sorted BCSCs or differentiated cancer cells derived from T47D cells. (D) Immunoblot showing CXCR1 and CXCR2 protein levels in matched mammosphere-enriched BCSCs and parental MCF-7 or T47D cells. (E) ALDEFLUOR assay of MCF-7 and T47D cells cultured with CM from BCSCs or parental cells. (F) MCF-7 or T47D cells were cultured with the CM derived from respective parental cells or mammosphere-enriched BCSCs in the presence or absence of 1 μg/ml IL8 neutralizing antibody and treated with a graded concentration of tamoxifen for 5 days. Cell viability was determined by MTT assay. (G) Immunoblot showing the IL8 protein levels in MCF-7 cells transfected with IL8 shRNA or vector. (H) MCF-7 cells were cultured with CM derived from parental cells or mammosphere-enriched shCONT-BCSCs or shIL8-BCSCs. (I) MCF-7 or T47D cells were cultured with the CM derived from respective parental cells or mammosphere-enriched BCSCs in the presence or absence of 100 nM AKT inhibitor IV (AIIV). (J) Kaplan–Meier plots of relapse-free survival (RFS) in ER-positive or TNBC breast cancer patients stratified according to their IL8 levels. The data was analyzed by Kaplan-Meier plotter software. All experiments were repeated at least three times. Results are shown as mean ± S.D. *P<0.05; **P<0.01; ***P<0.001; ns, not significant (Unpaired two-tailed Student’s t-test in Fig. S3A-S3E, others Two-way ANOVA test). **Fig. S4**. Targeting IL8 overcomes tamoxifen resistance. (A) Immunoblot showing the ICAM1, MIF and CXCL12 protein levels in MCF-7 TamR cells transfected with the respective shRNA or vector. (B) The MCF-7 TamR cells transfected with vector or shRNAs to ICAM1, MIF or CXCL12 were treated with a graded concentration of tamoxifen for 5 days. Cell viability was determined by MTT assay. (C) The statistical result of Fig. 4E. (D) The matched MCF-7 or MCF-7 TamR cells in the presence or absence of 100 nM AIIV were treated with a graded concentration of tamoxifen for 5 days. Cell viability was determined by MTT assay. (E) IHC staining of pAKT in MCF-7 TamR derived tumors treated with vector or repertaxin. Scale bar: 100 μm. (F-G) The statistical result of Fig. 4K (F) and Fig. 4M (G). (H-I) IHC staining of Ki-67 or TUNEL in tumor treated with Fulv+Palb with or without Repertaxin. Scale bar: 100 μm. All experiments were repeated at least three times. Results are shown as mean ± S.D. *P<0.05; **P<0.01; ***P<0.001; ns, not significant (Two-way ANOVA test in Fig. S4B and S4D, others one-way ANOVA followed by Tukey’s multiple comparison test). **Fig. S5**. BCSC-derived IL8 drives metastasis. (A) IL8 levels in breast cancer patients with different subtypes were analyzed in TCGA dataset. (B) The correlation between IL8 and EMT markers was analyzed in TCGA breast cancer dataset. (C) Transwell migration and invasion assay of MCF-7 and SUM159 cells transfected with IL8 shRNA or vector. Scale bar: 300 μm. (D) Transwell migration and invasion assay of MCF-7 and SUM159 cells cultured with CM derived from respective parental cells or mammosphere-enriched BCSCs in the presence or absence of 100 nM AIIV. Scale bar: 300 μm. (E) Transwell migration and invasion assay of MDA-MB-231 cells treated with 0.1 mM repertaxin. Scale bar: 300 μm. (F) Immunoblot analysis of CXCR1/2 levels in parental cells or BCSCs. (G) ALDEFLUOR assay of MDA-MB-231 cells cultured with recombinant IL8 or vehicle. (H) H&E staining of lung sections derived from tail vein injected MDA-MB-231 cells treated with vehicle or IL8. Scale bar: 300 μm. All experiments were repeated at least three times. Results are shown as mean ± S.D. *P<0.05; **P<0.01; ***P<0.001; ns, not significant (Unpaired two-tailed Student’s t-test). **Fig. S6**. β-CATENIN regulates the transcription of IL8. (A) MCF-7 cells were cultured with the CM derived from parental cells or mammosphere-enriched shCONT-BCSCs or shCTNNB1-BCSCs and treated with a graded concentration of tamoxifen for 5 days. Cell viability was determined by MTT assay. (B) Transwell migration and invasion assay of MCF-7 cells cultured with the CM derived from parental cells or mammosphere-enriched shCONT-BCSCs or shCTNNB1-BCSCs. Scale bar: 300 μm. (C) Schematic image of the regulation of BCSC-secreted IL8 in tamoxifen resistance and metastasis. All experiments were repeated at least three times. Results are shown as mean ± S.D. *P<0.05; **P<0.01; ***P<0.001; ns, not significant (Two-way ANOVA test in Fig. S6A, one-way ANOVA followed by Tukey’s multiple comparison in Fig. SB).

## Data Availability

The RNA sequencing data of MCF-7 cells have been deposited in GEO with the accession number: GSE156454. The RNA sequencing data of MDA-MB-231 cells have been deposited in GEO with the accession number: GSE22029. All other data supporting the findings of this study are available within the article and its supplementary data files.

## Abbreviations

CSCs: Cancer stem cells
BCSCs: Breast cancer stem cells
ER: Estrogen receptor
CM: Conditioned medium
GSEA: Gene set enrichment analysis
EMT: Epithelial-to-mesenchymal transition
TNBC: Triple-negative breast cancer
