# Supplementary material for: Paracrine secretion of IL8 by breast cancer stem cells promotes therapeutic resistance and metastasis of the bulk tumor cells
Source: Cell Commun Signal. 2023 Mar 13;21:59. doi: 10.1186/s12964-023-01068-6 (PMC10009947; doi:10.1186/s12964-023-01068-6)
Supplement: Supplementary file 2 — Additional file 1: Table S1. Sequences of shRNAs, and primers for cloning, ChIP assays, and qRT-PCR analysis. Table S2. List of antibodies used in western blot, immunohistochemistry and chromatin immunoprecipitation assay. [file 12964_2023_1068_MOESM2_ESM.docx]

**Supplementary Table 1 Sequences of shRNAs, and primers for cloning, ChIP assays, and qRT-PCR analysis.**

| shRNAs | Sense Strand (5'-3') | |
| --- | --- | --- |
| CXCL12  shRNA | CCGGTGTGCATTGACCCGAAGCTAACTCGAGTTAGCTTCGGGTCAATGCACATTTTT | |
| ICAM1  shRNA | CCGGCCGGTATGAGATTGTCATCATCTCGAGATGATGACAATCTCATACCGGTTTTT | |
| β-CATENIN  shRNA1 | CCGGAGGTGCTATCTGTCTGCTCTACTCGAGTAGAGCAGACAGATAGCACCTTTTTT | |
| β-CATENIN  shRNA2 | CCGGGCTTGGAATGAGACTGCTGATCTCGAGATCAGCAGTCTCATTCCAAGCTTTTT | |
| IL8  shRNA | CCGGCCGAACTTTAATTTCAGGAATCTCGAGATTCCTGAAATTAAAGTTCGGTTTTTG | |
| MIF  shRNA | CCGGGACAGGGTCTACATCAACTATCTCGAGATAGTTGATGTAGACCCTGTCTTTTTG | |
| Primers for plasmid construction | **Sense Strand (5'-3')** | **Antisense Strand (5'-3')** |
| Firefly luciferase cloning primer | CCGAATTCATGGCCGATGCTAAGAACATTA | AAGGATCCTTACACGGCGATCTTGCCGCCT |
| IL8 promoter cloning primer | TATAGCTAGCCCAGGCTAGTCTTGAACTCCTG | CCGGCTCGAGCATCTTTTCATTATGTCAGAGG |
| IL8 promoter mut primer | CCAAGTTCATTTCATTCATACCACATTTTGCCCACCATAT | CAAAATGTGGTATGAATGAAATGAACTTGGGTTTAAATCT |
| Primers for ChIP assay | **Sense Strand (5'-3')** | **Antisense Strand (5'-3')** |
| IL8 ChIP | ACGGGATCTCAGATTTTAGCA | TTGTAAGCCTGAACTAAGTTGT |
| GAPDH ChIP | TACTAGCGGTTTTACGGGCG | TCGAACAGGAGGAGCAGAGAGCGA |
| Primers for qRT-PCR | **Sense Strand (5'-3')** | **Antisense Strand (5'-3')** |
| GAPDH | TGCACCACCAACTGCTTAGC | GGCATGGACTGTGGTCATGAG |
| IL8 | GGTGCAGTTTTGCCAAGGAG | TTCCTTGGGGTCCAGACAGA |

**Supplementary Table 2. List of antibodies used in western blot,** **immunohistochemistry and chromatin immunoprecipitation assay.**

| Antibody | Assay | Origin | Dilution | Incubation period |
| --- | --- | --- | --- | --- |
| β-catenin | WB | #sc-7963, Santa cruz | 1:1000 | Over night |
| JUP | WB | #sc-8415, Santa cruz | 1:1000 | Over night |
| TJP1 | WB | #21773-1-AP, Proteintech | 1:1000 | Over night |
| OCLN | WB | #13409-1-AP, Proteintech | 1:1000 | Over night |
| Snail | WB | #sc-28199, Santa cruz | 1:1000 | Over night |
| FN1 | WB | #sc-8422, Santa cruz | 1:1000 | Over night |
| MMP2 | WB | #10373-2-AP, Proteintech | 1:1000 | Over night |
| VIM | WB | #sc-6260, Santa cruz | 1:1000 | Over night |
| IL8 | WB | #27095-1-AP, Proteintech | 1:1000 | Over night |
| p-AKT | WB | #9271s, CST | 1:1000 | Over night |
| t-AKT | WB | #sc-5298, Santa cruz | 1:1000 | Over night |
| p-AKT | IHC | #sc-7985-R, Santa cruz | 1:50 | Over night |
| ICAM1 | WB | #sc-8439, Santa cruz | 1:1000 | Over night |
| MIF | WB | #sc-271631, Santa cruz | 1:1000 | Over night |
| CXCL12 | WB | #17402-1-AP, Proteintech | 1:1000 | Over night |
| CXCR1 | WB | #sc-7303, Santa cruz | 1:1000 | Over night |
| CXCR2 | WB | #sc-7304, Santa cruz | 1:1000 | Over night |
| Actin | WB | #60008-1-Ig, Proteintech | 1:5000 | Over night |
| pERα S118 | WB | #2511, CST | 1:1000 | Over night |
| pERα S167 | WB | #42101, CST | 1:1000 | Over night |
| CCND1 | WB | #sc-8396, Santa cruz | 1:1000 | Over night |
| pRb | WB | #8516, CST | 1:1000 | Over night |
| Rb | WB | # 9309, CST | 1:1000 | Over night |
| TCF4 | ChIP | #sc-393407, Santa cruz | 1 μg | Over night |
| IL8 | Neutralizing | #ab18672, Abcam | 1 μg/ml |  |
