# Supplementary figures and images for: Paracrine secretion of IL8 by breast cancer stem cells promotes therapeutic resistance and metastasis of the bulk tumor cells
Source: Cell Commun Signal. 2023 Mar 13;21:59. doi: 10.1186/s12964-023-01068-6 (PMC10009947; doi:10.1186/s12964-023-01068-6)

Figure S1

A

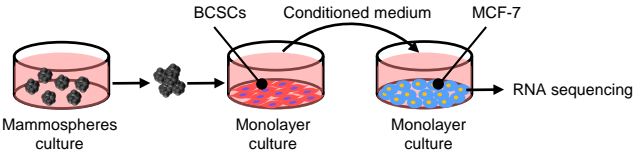

B

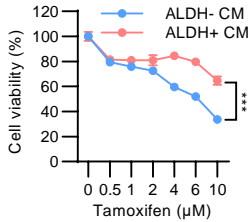

C

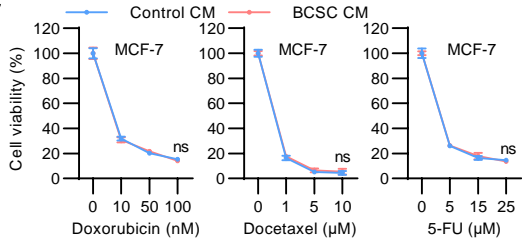

D

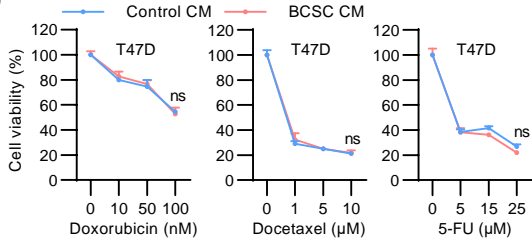

Figure S2

A

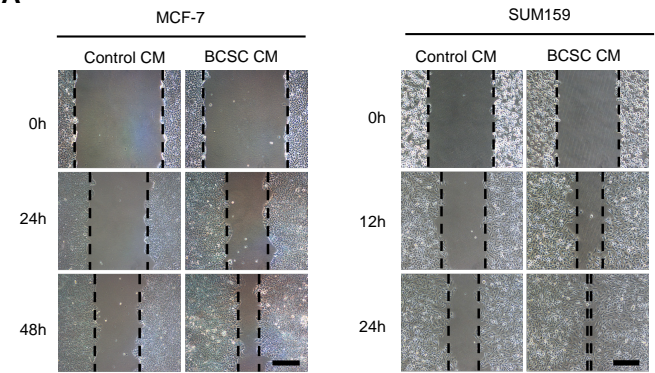

B

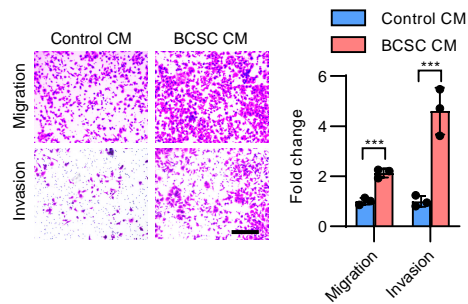

C

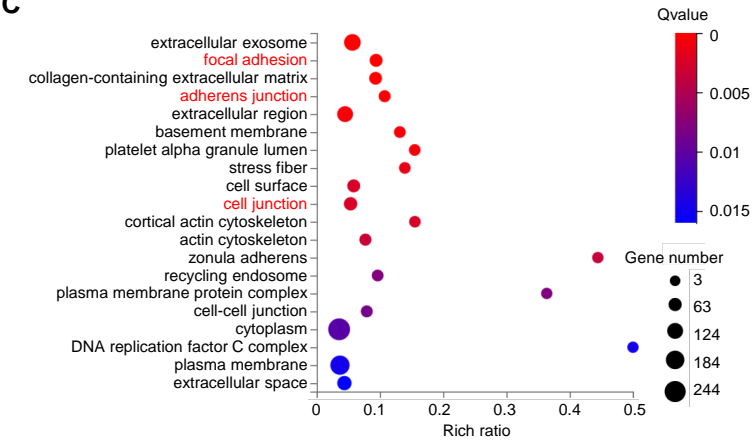

D

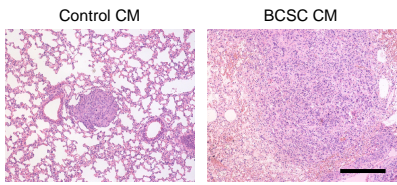

**Figure S3**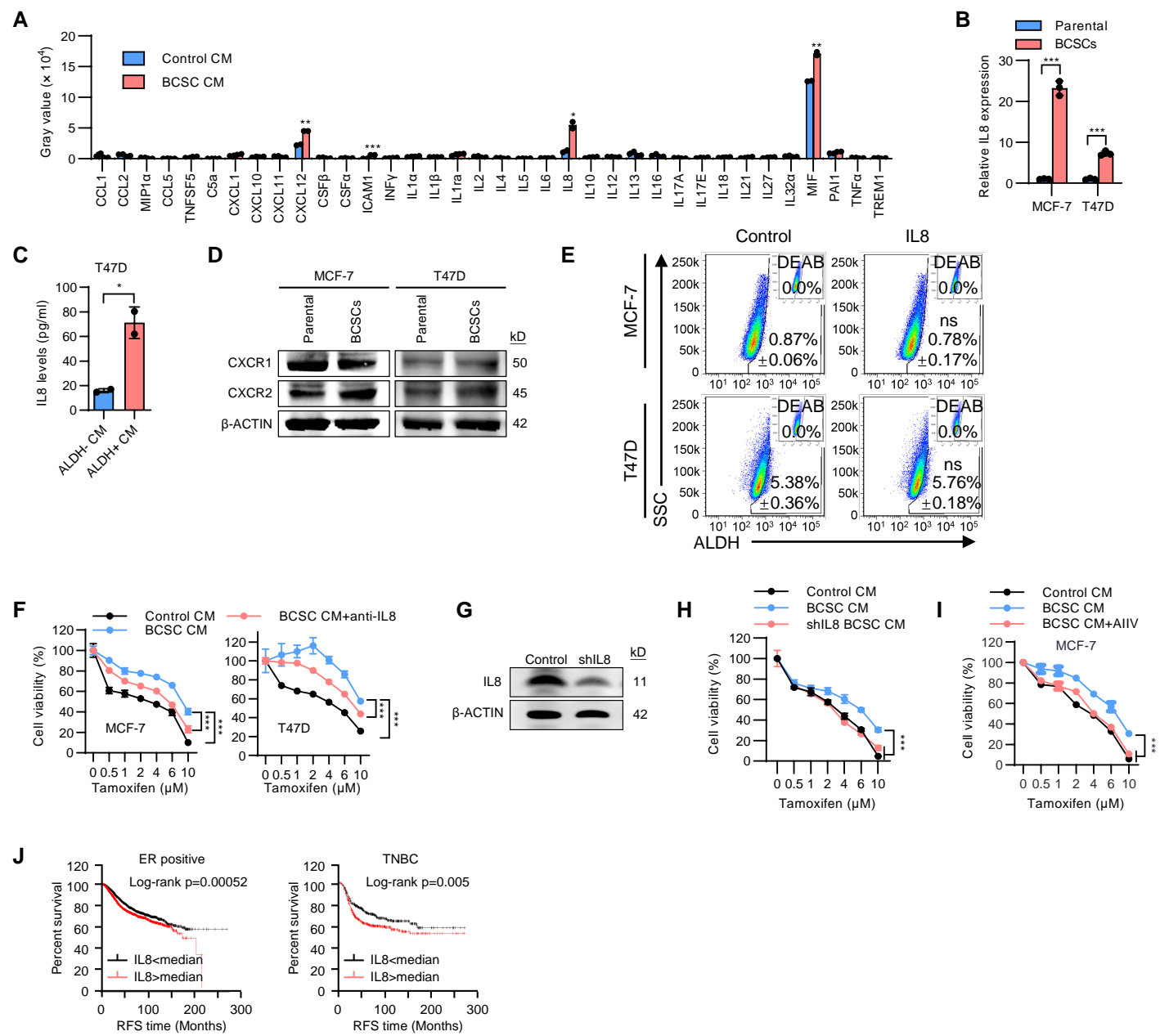

**Figure S4**

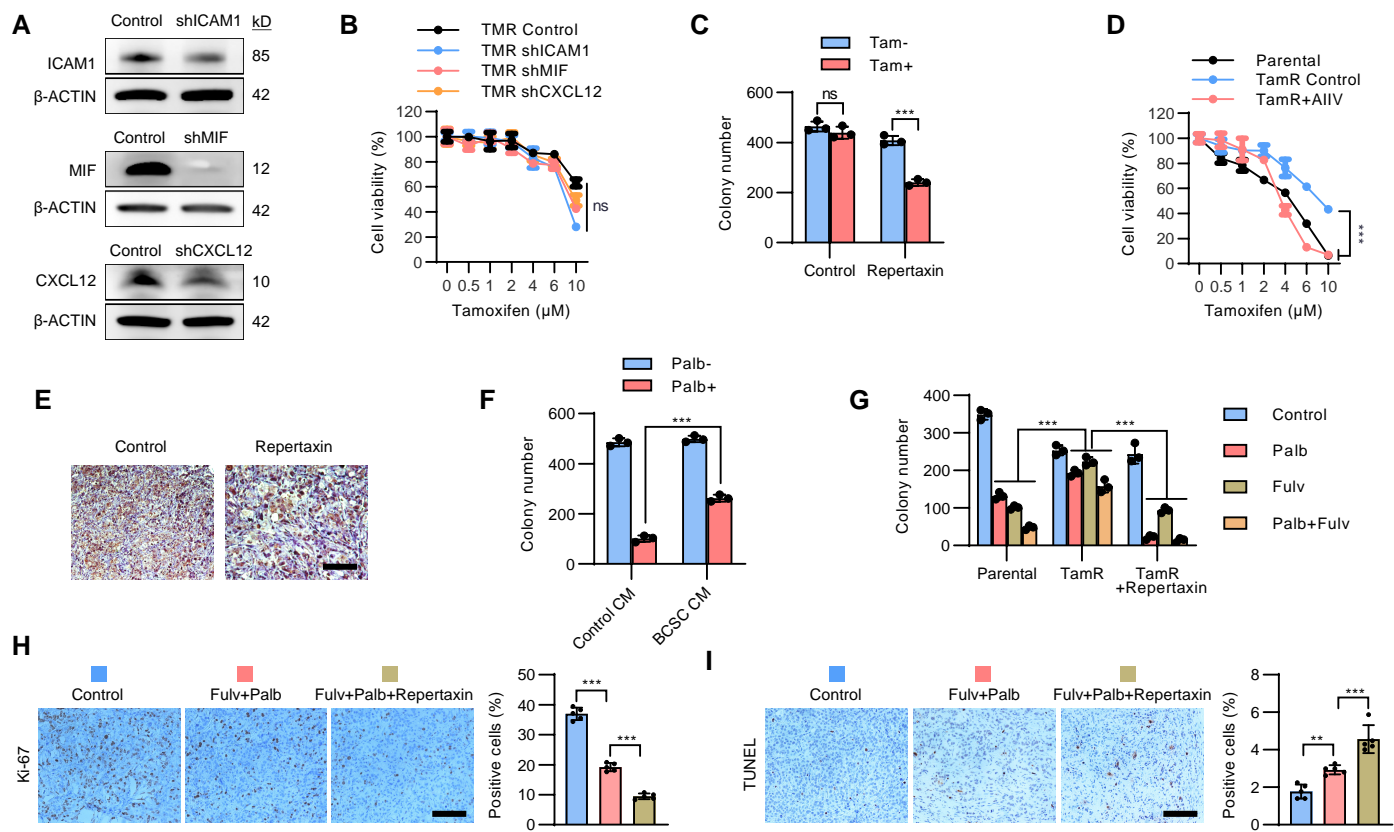

**Figure S5**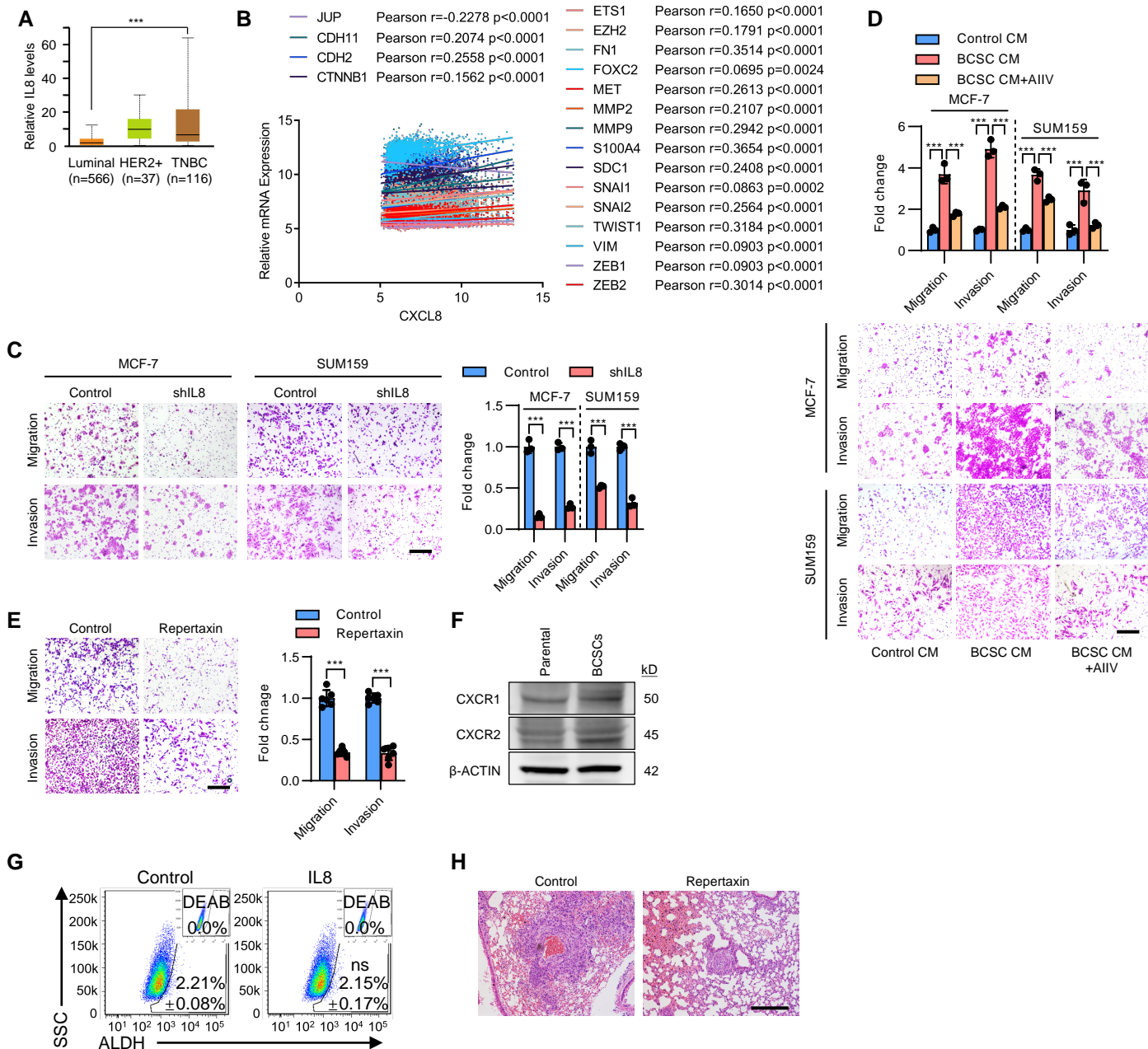

Figure S6

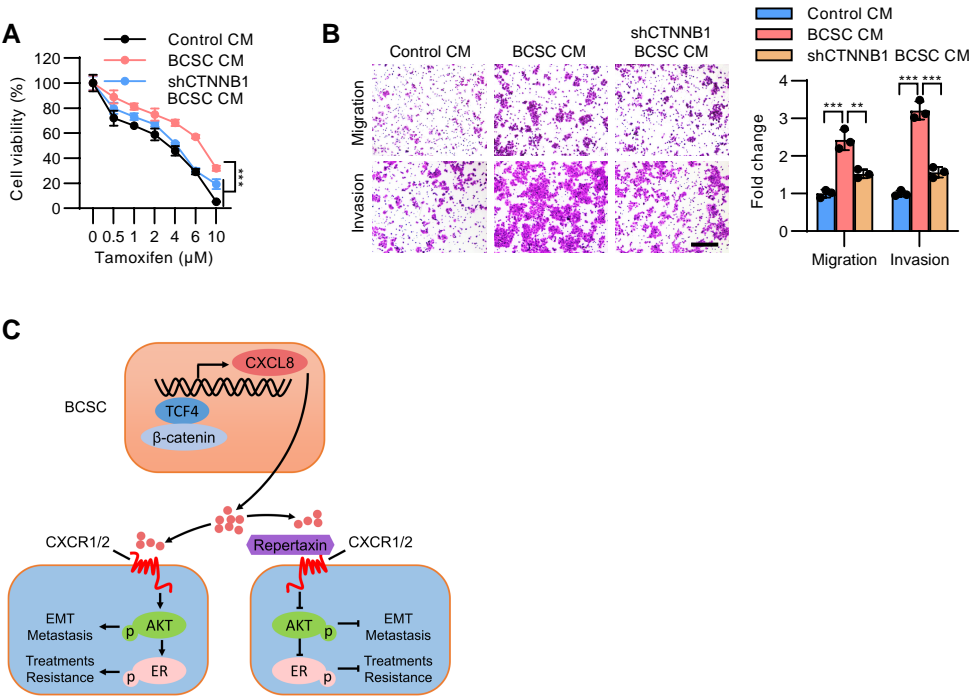

Supplement: Supplementary file 3 — Additional file 2: Fig. S1. BCSC secretome confers tamoxifen resistance. (A) The schematic of BCSC conditioned medium and RNA-sequencing. (B) T47D cells were cultured in CM derived from ALDEFLUOR assay sorted BCSCs or differentiated cancer cells and treated with a graded concentration of tamoxifen for 5 days. Cell viability was determined by MTT assay. (C-D) MCF-7 (C) or T47D (D) cells were cultured in CM derived from the respective mammosphere-enriched BCSCs or parental cells and treated with a graded concentration of doxorubicin, docetaxel or 5-FU for 5 days. Cell viability was determined by MTT assay. All experiments were repeated at least three times. Results are shown as mean ± S.D. *P<0.05; **P<0.01; ***P<0.001; ns, not significant (Two-way ANOVA test). Fig. S2. BCSC secretome confers metastasis. (A) In vitro wound-healing assay MCF-7 and SUM159 cells cultured with CM from parental cells or BCSCs. Scale bar: 300 μm. (B) Transwell migration and invasion assay of MDA-MB-231 cells cultured with CM from parental cells or BCSCs. Scale bar: 300 μm. (C) H&E staining of lung metastasis derived from MDA-MB-231 cells cultured with CM from parental cells or BCSCs. Scale bar: 300 μm. (C) GO analysis of genes enriched in MDA-MB-231 cells cultured with BCSC Cm compared to that with control CM. (D) H&E staining of lung sections derived from tail vein injected MDA-MB-231 cells cultured with control CM or BCSC CM. Scale bar: 300 μm. All experiments were repeated at least three times. Results are shown as mean ± S.D. *P<0.05; **P<0.01; ***P<0.001; ns, not significant (Unpaired two-tailed Student’s t-test). Fig. S3. BCSC-derived IL8 drives tamoxifen resistance. (A) Densitometric analysis of the protein array of factors present in CM derived from mammosphere-enriched BCSCs or parental MCF-7 cells. (B) qRT-PCR quantification of IL8 mRNA levels in matched mammosphere-enriched BCSCs and parental MCF-7 or T47D cells. (C) ELISA quantification of secreted IL8 protein levels in CM from [file 12964_2023_1068_MOESM3_ESM.pdf]
